# Not That Close to Mommy: Horizontal Transmission Seeds the Microbiome Associated with the Marine Sponge *Plakina cyanorosea*

**DOI:** 10.3390/microorganisms8121978

**Published:** 2020-12-12

**Authors:** Bruno F. R. Oliveira, Isabelle R. Lopes, Anna L. B. Canellas, Guilherme Muricy, Alan D. W. Dobson, Marinella S. Laport

**Affiliations:** 1 Laboratório de Bacteriologia Molecular e Marinha, Instituto de Microbiologia Paulo de Góes, Universidade Federal do Rio de Janeiro, Rio de Janeiro 21941902, Brazil; bfroliveira@micro.ufrj.br (B.F.R.O.); rodrigueslopes.isabelle@gmail.com (I.R.L.); annaluizabcc@gmail.com (A.L.B.C.); 2 School of Microbiology, University College Cork, T12 Y960 Cork, Ireland; a.dobson@ucc.ie; 3 Laboratório de Biologia de Porifera, Museu Nacional, Universidade Federal do Rio de Janeiro, Rio de Janeiro 20940040, Brazil; muricy@mn.ufrj.br; 4 Environmental Research Institute, University College Cork, T23 XE10 Cork, Ireland

**Keywords:** 16SrRNA gene-diversity analyses, Homoscleromorpha, marine bacteria, microbial transmission, *Plakina*, sponge microbiology

## Abstract

Marine sponges are excellent examples of invertebrate–microbe symbioses. In this holobiont, the partnership has elegantly evolved by either transmitting key microbial associates through the host germline and/or capturing microorganisms from the surrounding seawater. We report here on the prokaryotic microbiota during different developmental stages of *Plakina cyanorosea* and their surrounding environmental samples by a 16S rRNA metabarcoding approach. In comparison with their source adults, larvae housed slightly richer and more diverse microbial communities, which are structurally more related to the environmental microbiota. In addition to the thaumarchaeal *Nitrosopumilus*, parental sponges were broadly dominated by Alpha- and Gamma-proteobacteria, while the offspring were particularly enriched in the Vibrionales, Alteromonodales, Enterobacterales orders and the Clostridia and Bacteroidia classes. An enterobacterial operational taxonomic unit (OTU) was the dominant member of the strict core microbiota. The most abundant and unique OTUs were not significantly enriched amongst the microbiomes from host specimens included in the sponge microbiome project. In a wider context, *Oscarella* and *Plakina* are the sponge genera with higher divergence in their associated microbiota compared to their Homoscleromorpha counterparts. Our results indicate that *P. cyanorosea* is a low microbial abundance sponge (LMA), which appears to heavily depend on the horizontal transmission of its microbial partners that likely help the sponge host in the adaptation to its habitat.

## 1. Introduction

Microbial transmission is absolutely essential for the existence of the holobiont unit, which is defined as the eukaryotic host and its associated microbial communities [1]. Its importance is such that two of the four principles of the hologenome theory of evolution address the transmission patterns of microbial symbionts throughout the host lifetime [2,3]. Essentially, they span along a continuum of two opposite modes: vertical transmission (VT), in which symbionts are transmitted from the parental tissues to the offspring normally through the germline; and horizontal transmission (HT), where the environment serves as the source of free-living microorganisms [4,5]. Exclusive vertical transmitted symbiosis tightly links the symbionts to the evolutionary path of the host, while increasing their specialization towards total dependence on the host. In contrast, HT allows the acquisition of locally-advantageous and novel microbial partners, although it may leave the host more susceptible to the attack by predators and lacks the symbiont assurance guaranteed by VT [6,7,8]. A mixed model couples both VT and HT modes and would be the most fitting strategy for the majority of host-microbe interactions. Theoretically, this hybrid model achieves a trade-off balance between these antithetic modes and results in a dynamic change in the microbial composition between generations [9,10].

Sponges are amongst the oldest and most highly successful examples of animal-microbe symbioses which thrive in marine habitats [11]. To date, these filter-feeding invertebrates are reported to host more than 50 prokaryotic phyla, which play essential roles in a number of important functions, ranging from involvement in biogeochemical cycles to the production of metabolites that function in host defense [12,13]. Earlier microscopy and microbiology-based work indicated vertical inheritance as a fundamental mechanism in preserving key and specific microbial members within the same sponge species or sponges, in general. However, despite the fact that “sponge microbial specificity” alludes to a primary role for VT, the presence of the same microbial phylotypes in hosts located in distant geographic locations also pointed to the important role played by environmental capture [14,15]. With the increased application of next-generation sequencing (NGS) as the general approach in the characterization of sponge-associated microbial communities, it became clearer that the role that HT was playing in the establishment of sponge microbial community structures had been severally underestimated. From initial observations indicating the possibility of seawater as a “microbial seedbank” for sponges [16] to more recent studies supporting environmental acquisition as a major contributor to a functional sponge microbiome [17,18,19], HT has been progressively occupying a more central role, while the primary importance of VT has been called into question [20]. The replacement of “sponge-specific microbial clusters” [21] by the term “sponge-enriched microbial clusters” [22,23] together with the proposal of mechanistic models involving interplay between both VT and HT modes [24,25,26,27] are indications of how these changing views are progressively converging into the concept of a mixed mode of microbiome transmission for the sponge holobiont. Nevertheless, more studies are required to shed more light on the microbial transmission dynamics in a wide array of species, particularly in underexplored sponge taxa.

Homoscleromorpha, the youngest lineage in the phylum Porifera, constitutes a group of sponges whose microbial ecology has as yet been poorly studied. Driven by molecular phylogenetic analyses within an integrative taxonomy approach [28], Homoscleromorpha was elevated from a former family or suborder within the Demospongiae class to a new class status at the beginning of the decade [29]. In addition to a number of unique morphological and developmental traits that place these sponges at an evolutionary junction split between Porifera and the remaining metazoans [30,31,32,33], Homoscleromorpha are known to include marine sponges with reduced sizes, smooth surfaces, and with irregular anatomy [34]. A common ecological feature of these sponges is a preference for shadowed or low-light zones, such as the undersides or boulders of rocky and coralline habitats and, especially, semi-dark or dark submarine caves [35,36]. Taxonomically, two families, Oscarellidae and Plakinidae, constitute the major branches within the only order in this class (Homosclerophorida), with the former harboring two genera, *Oscarella* and *Pseudocorticium*, and the latter, seven genera, *Plakina*, *Plakortis*, *Plakinastrella*, *Corticium*, *Placinolopha*, *Tetralophora* and *Aspiculophora*. In total, 126 homoscleromorph species exist, representing 1.35% of the worldwide poriferan diversity [37].

The genus *Plakina* has the highest number of Homoscleromorpha species that have to date been described, involving a total of 39 species [37]. Currently recognized as true cosmopolitans, new *Plakina* species have recently been found in cryptic habitats, such as in Caribbean [38,39], Mediterranean [40] and Pacific [41,42] caves. In particular, taxonomic reports have demonstrated that the Tropical Western Atlantic coast is a striking biodiversity hotspot for new *Plakina* species, which run the risk of extinction prior to their complete characterization [34,43,44]. This has led to the description of two new *Plakina* species, *Plakina cabofriense* and *Plakina cyanorosea*, in a tide pool near to the touristic coastal zone of the Cabo Frio city (Rio de Janeiro state, Brazil). Both sponges are relatively small, with a thinly encrusting colorful macromorphology, and were frequently encountered attached to rocks burrowed in the sandy sediment and cytological evidence indicated the presence of diverse microbial morphotypes [45].

To increase the knowledge base on the microbiology of Homoscleromorpha, we have focused on characterizing the cultivable microbiota associated with these marine sponges [46], with a recent focus on the diversity and antimicrobial potential of bacteria isolated from these new *Plakina* species isolated from this tidal pool environment [47,48]. By targeting the V4 region of the 16S rRNA gene, we here report on the bacterial and archaeal communities in different life stages of the marine sponge *P. cyanorosea* and compare them with the communities present in the surrounding seawater and sediment present in the sponges’ shallow-water ecosystem. To unveil differences in the microbiota associated with Homoscleromorpha sponges, it was conducted a comparative global analysis between the sponges from this class included in the Sponge Microbiome Project (SMP) and the samples herein studied. Possible enrichment in sponge-associated microbial lineages in these plakinid life stages and the environmental samples was assessed by direct BLAST searches against the Sponge Earth Microbiome Project (EMP) database. The current study follows on from a recent report about the microbiota associated with adult samples of *Plakina kanaky*, a cave-inhabitant polychromatic and HMA sponge [49]. Without assessing the microbiota of any developmental stage, the authors proposed that a “Candidatus *Entotheonella*” OTU had been vertically transmitted in *P. kanany* [49]. Our study suggests that *P. cyanorosea* is a low microbial abundance (LMA) sponge, which apparently evolved to capture environmental microorganisms in its original shallow-water habitat. Up to now, this is the first work about the prokaryotic microbiota associated with *P. cyanorosea* and the transmission of microbiota in a *Plakina* sponge.

## 2. Materials and Methods

### 2.1. Collection and Processing of the Samples

**Figure 1:**
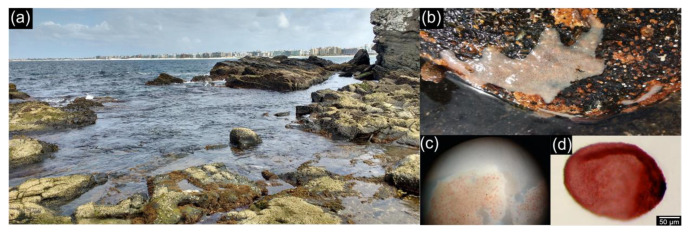
Sampling site and *P. cyanorosea* adult and larvae: (**a**) “Carolina Tidal Pool” (22°53′13.0′′ S, 042°00′17.9′′ W) under low-tide conditions (30-cm depth). Cabo Frio city can be seen on the horizon in the right-center side of the picture; (**b**) external morphology of *P. cyanorosea* adult on the under-surface of a small rock collected at the tidal pool; (**c**) closer examination under a dissecting microscope of a *P. cyanorosea* adult specimen with the larvae concentrated at the sponge base; (**d**) a free-living larvae of *P. cyanorosea* under a light microscope.

The sampling of sponge adults, seawater and sediment was conducted in a shallow tide pool near the entrance of the Itajurú Channel (“Carolina Tidal Pool”; 22°53′13.0″ S, 042°00′17.9″ W) situated in the coastal line of the city of Cabo Frio (Rio de Janeiro state, Brazil) at 30-cm depth under low-tide conditions (Figure 1a) in May 2017. Five specimens of *P. cyanorosea* (*n* = 5), 6 to 7 cm wide, were aseptically collected from the under-surfaces of small rocks (Figure 1b). They were subsequently washed with sterilized artificial seawater (ASW, g/L: NaCl 23 g, MgSO_4_·7H_2_O 4.93 g, MgCl_2_·6H_2_O 4.07 g, CaCl_2_·H_2_O 1.47 g, KCl 0.75 g, NaHCO_3_ 0.17 g) to remove the excess of sediment and loosely-attached microbiota, and then finally placed into ASW vials. The pink morphotype of the sponge was chosen to facilitate the posterior recovery of the dark reddish larvae (50–200 μm) [45], which are normally visible just beneath the sponge surface (Figure 1c,d). Five 2.0-L sterilized flasks were filled with ambient seawater from different points of the tide pool, all from the same vicinity (2–5 m) of the collected adult specimens. Five sediment samples were also obtained from the same vicinity, at a minimum depth of 10 cm, and were stored ziplocked plastic bags. Each of the five seawater and sediment samples was collected nearby to each of the five representative adult specimens. All the samples were maintained on ice in a coolbox and immediately transferred to the lab for further processing.

Larvae recovery was carried out by using a stereolupe (Zeiss, Oberkochen, Germany) under aseptic conditions. Following a new washing step with sterilized ASW, each one of the adult specimens was transferred to 15 mL ASW filled Petri dishes (90 × 15 mm). With the aid of autoclaved forceps, the sponge was gently agitated to release the larvae from their inner surface. Upon visualization of at least 5 to 10 free larvae with no signs of disruption in the ASW suspension, these larvae were then placed in 1 mL of CHAOS solution (guanidine thiocyanate 4 M, N-lauryl sarkosyl 0.5%, Tris 25 mM pH 8.0, 2-mercaptoethanol 0.1 M) [50] using disposable Pasteur pipettes. Once the larvae were successfully recovered, the parental sponges were subsequently transferred into 1.0 mL of CHAOS solution.

Seawater samples were processed in a two-step sequential filtration step, firstly by employing a 0.8 μm-pore-size filter and then a 0.22 μm-filter, with these filter membranes being placed in CHAOS solution. A total of 1.0 kg of each sediment sample were initially sifted in an ethanol-disinfected sieve (mesh size 0.25 mm) and one gram of the sieved sediment was resuspended in CHAOS solution. Following sampling, a total of three representative adult specimens (A1, A2 and A3), their respective larvae pool (L1, L2 and L3), together with the filter membranes, corresponding to the surrounding seawater (W1, W2 and W3), and sediment samples (S1, S2 and S3) were subsequently processed. All these samples were maintained in CHAOS solution at 4 °C until DNA extraction [50].

### 2.2. DNA Extraction and Sequencing

A modified phenol-chloroform method [50] was used for the isolation of metagenomic DNA. Prior to DNA extraction, the 0.22 μm filter membranes were clipped in pieces using 70% ethanol-sterilized surgical scissors and resuspended again in the same CHAOS solution. DNA extraction was performed in triplicate for each sample. The quality of the extracted DNA was assessed by 0.8% (*w*/*v*) agarose gel electrophoresis. The DNA concentration and purity were estimated at a wavelength of 260 nm, considering the 260/230 and 260/280 ratios using a NanoVue Plus spectrophotometer (GE Healthcare, Chicago, IL, USA). All DNA samples were frozen at −20 °C until subsequent analysis. Following a purification step with magnetic beads, the metagenomic DNA was PCR amplified using the universal bacterial/archaeal primers 515F (5′-GTGCCAGCMGCCGCGGTAA-3′) and 806R (5′-GGACTACHVGGGTWTCTAAT-3′), which target the V4 region of the 16S rRNA gene sequence in accordance with the Earth Microbiome Project (EMP) protocols [51,52]. A multiplex identifier barcode was attached to the forward primer to barcode the samples. Amplifications were performed in a 20 μL total reaction volume with: 0.3 μM of each universal primer, 1× buffer GoTaq Colorless Master Mix (Promega, Madison, WI, USA) and 20 ng of metagenomic DNA. Cycling conditions were adjusted to the following program: 94 °C for 3 min, then 29 cycles of 94 °C for 45 s, 50 °C for 1 min, and 72 °C for 1 min, and a final extension of 72 °C for 1 min 30 s. Amplicon integrity was confirmed by 2.0% (*w*/*v*) agarose gel electrophoresis. Triplicate PCR reaction products were combined, purified using Agencourt AMPure XP kit (Thermo Fisher Scientific, Waltham, MA, USA) with a bead volume: PCR product ratio of 4:5 in accordance with the 16S Metagenomic Sequencing Library Preparation for the Illumina MiSeq system. Real-time PCR was applied for quantification, in agreement with the manufacturer’s protocol KAPA-KK4824 (Library Quantification Kit-Illumina/Universal, Kapa Biosystems). After normalizing all samples at a concentration of 2 nM, an equimolar pool of DNA was sequenced on an Illumina MiSeq 2500 platform (Illumina^®^ Sequencing, San Diego, CA, USA). Sequencing was performed at BPI (Biotecnologia, Pesquisa e Inovação, Botucatu, São Paulo state, Brazil).

FASTQ files containing the DNA sequences were used for downstream computational analyses. Illumina sequencing data have been deposited as an NCBI BioProject PRJNA663922.

### 2.3. Bioinformatic Analyses

Illumina sequence reads were processed and analyzed using mothur v. 1.44.0 software [53] and the R packages phyloseq v. 1.5.21 [54] and microbiome v. 2.1.26 [55]. Briefly, quality-filtered and demultiplexed forward and reverse paired-end files were joined into contigs and screened to eliminate sequences <250 bp, containing any ambiguous bases and excessively long homopolymers. Following this screening step, redundant sequences were removed and then aligned to a modified version of the SILVA database (release 138, mothur-formatted), which was previously subjected to a virtual PCR with the universal primer set 515F-806R used for Illumina sequencing [56]. The resulting alignment was screened and filtered for size adjustment and elimination of uninformative columns of the alignment. The sequences were preclustered (parameters diffs = 1), followed by chimera detection and removal. Taxonomic assignment was conducted by classifying the sequences against a modified version of the RDP database with a bootstrap cutoff of 80 (release 16, mothur-formatted) [57]. The RDP database was also processed by a virtual PCR with the sequencing primers as performed for the SILVA database in the alignment step. Non-target sequences, such as those from chloroplasts, mitochondria and Eukarya, were removed. Pairwise distances between aligned sequences were calculated, followed by their clustering into operational taxonomic units (OTUs) by using a cutoff of 3% of dissimilarity, with the removal of eventually-produced singleton sequences. To avoid artefacts due to sampling effort on subsequent diversity calculations, each sequence dataset was subsampled to the lowest read count.

Alpha-diversity metrics were estimated by taking into account the number of OTUs and the Chao1 species estimator [58], both used for community richness, and the Shannon index [59] for community diversity. Community composition summaries (phylum and class levels) and the list of the top 30 OTUs (representing at least 20% of all reads in at least one of the samples) were plotted in R as a barplot and heatmap, respectively. Representative sequences of each OTU were obtained in Mothur (“get.oturep” command) for downstream OTU-level analyses.

Unique OTUs in both sponge life stages (adult and larvae) and the environmental samples (seawater and sediment) as well as OTUs uniquely shared across selected groups (adults and larvae, water and sediment, adults and environmental samples, larvae and environmental samples) were obtained using the “get.sharedseqs” command in mothur, setting the “uniquegroups” and “sharedgroups” parameters, respectively. The core microbiota, corresponding to the fraction of OTUs detected in all samples at a minimum of 0.01% abundance and 95% prevalence, was calculated following the core microbiota analyses workflow from the microbiome R package. The “get.coremicrobiome” command in mothur was applied to derive the core microbiota, the fraction of OTUs that are found in each group (adults, larvae) and between combinations of these groups (adults vs. larvae, adults vs. environmental sample groups, larvae vs. environmental sample groups) at a minimum of 0.001% abundance and 100% prevalence (OTUs strictly present in all samples).

### 2.4. Statistical Analyses

To visualize the structure of microbial communities at the OTU level, non-metrical multidimensional scaling (NMDS) based on the Bray–Curtis similarity matrices was computed using the PAST v. 4.0.3 software [60]. Subsequently, the normality tests in PAST, statistical differences between the diversity and richness indexes were assessed with the one-way analyses of variance (ANOVA) for all groups, with the application of the post hoc Tukey’s test to confirm which sample group was significantly different from each other. Pairwise comparisons for sponge samples (adult and larvae) vs. environmental samples (seawater and sediment) and between adult and larvae samples were checked using the *t*-test.

Given the Bray–Curtis OTU similarity matrices, one-way permutational multivariate analyses of variance (PERMANOVA) was performed to evaluate statistical differences (*p* < 0.05) in the overall OTU assemblages between the groups of samples, with factor source (adults, larvae, seawater and sediment) being the only variable of interest. Permutational multivariate analyses of dispersion (PERMDISP) were subsequently conducted to identify differences in homogeneity (dispersion) among groups for all significant PERMANOVA results. These multivariate statistics were computed in R, using the adonis2 and betadisper functions from the vegan 2.5–6 package [61].

### 2.5. Analyses of Sponge-Enriched OTUs

Representative sequences of the top30 dominant OTUs and also the seven unique OTUs (five in sponges, two in environmental samples) and the six shared OTUs across selected cross-groupings were selected to detect which OTUs from these samples were significantly enriched in the 3569 sponge specimens (representing 269 sponge species) from the Sponge Microbiome Project (SMP) database [62]. For that, we followed a previous analysis protocol [63], but the RDP database (release 16) was applied for taxonomic classification since it was used for the taxonomic assignment of samples studied herein. Initially, a curated reference sponge microbiome reference database with 75,756 high-quality deblurred OTUs was produced based on the available data (final BIOM table) from the Sponge Earth Microbiome Project (EMP) (https://github.com/amnona/SpongeEMP). Then, the selected query sequences were submitted to local BLASTn [64] searches (NCBI-BLASTN-2.10.0+) against this created reference database. The sponge microbiome deblurred OTUs sequences with 100% of identity to any of 43 selected query sequences were uploaded to the sponge EMP online server (www.spongeemp.com) to identify possible OTUs enriched in any of the host specimens constituting the SMP database. Only hits for sponge hosts included in the sponge EMP were considered in the enrichment analyses.

### 2.6. Global Analyses of Homoscleromorpha-Associated Prokaryotic Microbiota

To place the microbiome of the different *P. cyanorosea* life stages samples into a broader context, we compared them with the microbiota of other Homoscleromorph sponges, by employing a previous analyses workflow [65]. Briefly, the 16S rRNA sequence data of 88 Homoscleromorph sponges, included in the SMP database (Table S1), was directly downloaded from the SRA database. mothur v. 1.44.0 and the R packages phyloseq and microbiome were also employed for these global analyses.

Initially, all FASTA files were merged and aligned as previously mentioned. Given that the V4 region of the 16S rRNA gene from the sponges within the SMP database were shorter fragments (150 bp) due to the employed sequencing platform (Illumina HiSeq 2500), the aligned sequences were trimmed to this exact 150-bp long overlapping region; and those sequences aligned to the delimited position were then exclusively maintained [66]. The sequence data was preclustered, submitted for chimera removal and classified against the RDP reference database, with a bootstrap cutoff = 80. After the elimination of mitochondria, chloroplast and Eukarya sequences, pairwise distances were computed, and the OTU classification was performed under a cutoff of 3% dissimilarity and singletons were removed from the final sample dataset. Rare taxa representing <1.0% of relative abundance of the microbial communities were aggregated for composition-level analyses.

### 2.7. Molecular Sponge Identification

Adult sponge specimens were identified based on amplification and sequencing of conserved marker genes encoding for the cytochrome oxidase subunit I (*cox*-1) and the 28S ribosomal RNA (rRNA) region. PCR amplification for the *cox*-1 and 28S rRNA genes were performed as previously detailed [67,68], respectively. The amplicon size and quality were checked by 2.0% (w/v) agarose gel electrophoresis using a Low DNA Mass Ladder (Thermo Fisher Scientific, Waltham, MA, USA) as standard. DNA quantity and purity were assessed spectrophotometrically, as previously mentioned. The PCR products were submitted to Sanger sequencing in a 3500 Genetic Analyzer (Applied Biosystems, Foster City, CA, USA). The generated *cox*-1 and 28S rRNA sequences were quality-inspected and edited within the interface of the Sequence Scanner 2 software. The NCBI BLASTn [63] searches were performed for the identification of the closest relatives deposited in the GenBank database. All produced sequences were submitted to the GenBank nucleotide database under the accession numbers MT750257, MT750258 and MT750260 (*cox*-1) and MT742287, MT742287 and MT742290 (28S rRNA).

## 3. Results

### 3.1. Molecular Sponge Taxonomy

The *P. cyanorosea* specimens selected for the microbiota characterization were identified based on the sequencing of the recommended marker genes, *cox*-1 and 28S rRNA, for sponge molecular identification [68]. Following BLASTn analyses with the mitochondrial gene, all sponge samples had as the closest relatives the *cox*-1 sequences from *P. cyanorosea* (KY421460.1, KY421463.1, KY421464.1, KY421465.1) at a 99–100% nucleotide identity, succeeded by other species from the *Plakina*, *Plakortis*, *Plakinastrella* and *Corticium* genera at a 91–93% nucleotide identity. BLASTn analyses with the ribosomal marker revealed that 28S rRNA sequences from the *Plakinastrella* (KC869521.1, AY561869.1 AY561870.1) and *Plakortis* (KC869521.1) genera were the top hits at a 96–97% nucleotide identity. We believe this result may derive from the few *Plakina* 28S rRNA gene sequences that are currently deposited in the NCBI GenBank Database, which is comprehensible since this marker has been recently adopted for taxonomic purposes. In addition, *Plakina* is a Homoscleromorpha genus whose taxonomy is likely to be changed in the coming years, with the potential division of the genus into new genera in association with the broader reclassification of other Plakinidae [38].

### 3.2. Prokaryotic Community Composition

**Figure 2:**
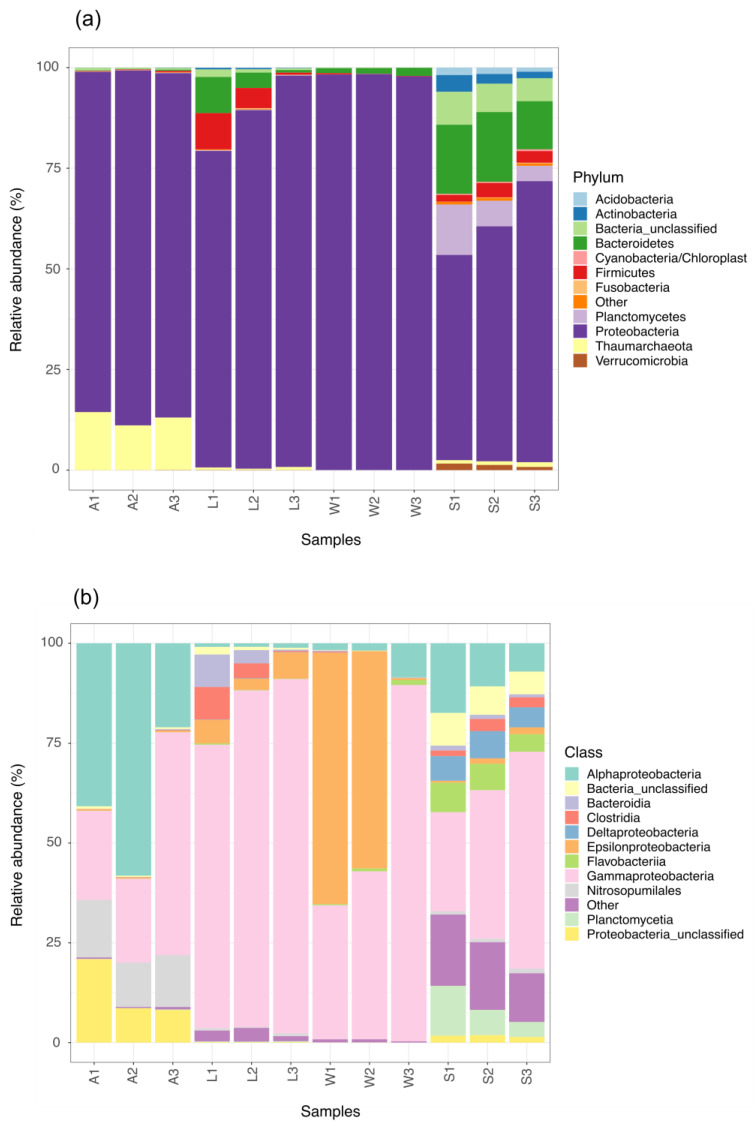
Relative abundance of prokaryotic community composition in the different life stages of the marine sponge *P. cyanorosea* and the ambient seawater and sediment: (**a**) Phylum level; (**b**) Class level. Microbial taxa with an abundance level <1.0% of total composition were depicted together (“Other”). A: adults; L: larvae; W: seawater; S: sediment.

In total, 3,845,463 reads, of 250 bp in length on average, were obtained after demultiplexation, quality filtering and downstream processing (Table S2). Subsequently to taxonomic classification and subsampling to the smallest read count, these sequences yielded 27,231 OTUs (Table S2) at a 97% identity. The domain Bacteria accounted for 97% of the total OTUs, with the remaining 3.0% being Archaea. Thirty different bacterial and five archaeal phyla were identified, with the Proteobacteria being the predominant phylum. Bacteroidetes and Firmicutes were the second and third most abundant bacterial phyla respectively, being particularly enriched in the microbial communities of larvae and the environmental samples. Thaumarchaeota was the dominant archaeal phylum, particularly in the adults, where the thaumarchaeal mean abundance was 9.0% (Figure 2a). Other detected archaeal phyla, such as Woesearcheota, Parcearchaeota and unclassified Archaea, were concentrated in the sediment samples, but at a very low relative abundance (<0.01%). Moreover, up to one-fifth of the sediment microbial communities were unclassified Bacteria, while the abundance for this unresolved rank varied from 2.0 to 5.0% throughout the other samples.

At the class level, Alpha- and Gamma-proteobacteria made up to 70% of the microbial community composition in the sponge parental samples. Between 27% to 40% of the adult OTU were unclassified Alphaproteobacteria. Focusing on the larvae microbiota, the abundance of the Gammaproteobacteria accounted for 75 to 90% of the overall community (Figure 2b), in particular from the orders Alteromonodales, Enterobacterales and Vibrionales. In addition, members from the Clostridia, Bacteroidia and Epsilonbacteria classes were found at a mean relative abundance of 5.6%, 5.0 and 5.3% in the offspring symbiont communities, respectively. Two of the three ambient seawater samples were dominated by Gamma- and Epsilon-proteobacteria, which together comprised nearly 75% of the microbial communities in these samples (Figure 2b). Notwithstanding this, the highly-diverse microbiota of sediment was predominantly represented by Gamma-, Alpha-, Delta-proteobacteria, followed by the class Planctomycetia and several classes from the Bacteroidetes phylum.

### 3.3. Prokaryotic Community Diversity and Structure

**Figure 3:**
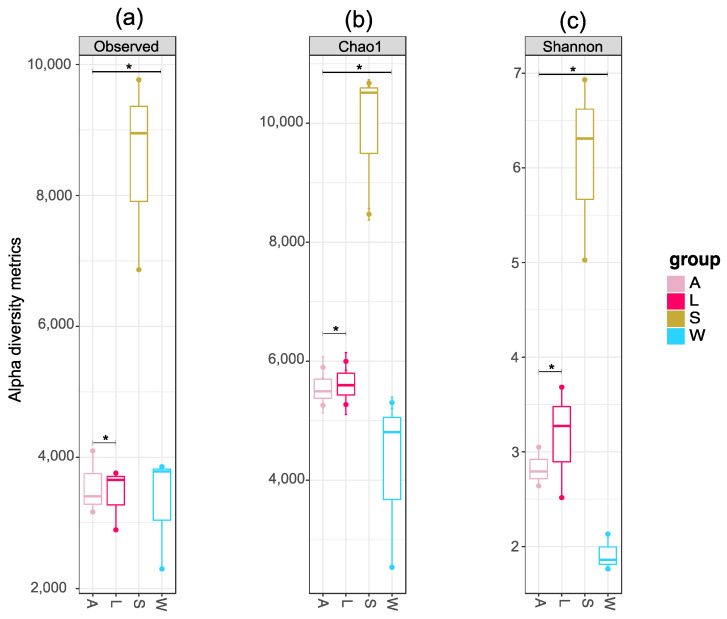
Boxplots of the alpha diversity metrics of the prokaryotic microbiota found in different life stages of the marine sponge *P. cyanorosea* and the ambient seawater and sediment: (**a**) Number of operational taxonomic units (OTUs); (**b**) Chao1 species estimator; (**c**) Shannon index. * *p* < 0.05 (all samples—ANOVA test and post hoc Tukey’s test; pairwise comparisons—*t*-test). A: adults; L: larvae; W: seawater; S: sediment.

The specific value counts obtained for each alpha diversity metrics are presented in Table S2. The number of OTUs ranged from 2304 (W1) to 9787 (S3). The Chao1 estimator also varied considerably, from 2533.738 (W3) to 10,680.7 (S1). Overall, the number of OTUs in sediment samples varied from 6867 (S1) to 9787 (S3), while this OTU count did not surpass 5000 in the remaining sample groups. (Figure 3a,b). Likewise, the Shannon indexes in seawater samples fluctuate from 1.765604 (W1) to 2.132883 (W2), while this diversity metric were numerically superior in the samples included in other groups (Table S3). Larval microbial communities were slightly more diverse compared to their respective adults, with the Shannon index calculated for the sediment samples being more than double the values obtained for the larvae samples (Figure 3c). One-way ANOVA testing was applied to the alpha-diversity means, as means to verify the existence of statistical differences (*p* < 0.05) between the sample groups. Post hoc Tukey’s test confirmed that the adults, larvae and seawater samples were significantly different from the sediment samples (*p* = 0.004). Pairwise comparisons (*t*-test) also demonstrated significant differences between the richness and diversity of microbial assemblages in the sponge life stages and environmental samples (OTU number, *p* = 3.2751 × 10^−5^; Chao1 index, *p* = 1.7522 × 10^−6^; Shannon index, *p* = 1.9968 × 10^−5^). These differences were also detected between adults and larvae microbiota (OTU number, *p* = 5.5812 × 10^−6^; Chao1 index, *p* = 5.7601 × 10^−8^, Shannon index, *p* = 1.3548 × 10^−5^) (Figure 3; Table S3). Rarefaction curves reached near saturation for most of the samples, excepting one larva (L3) and one seawater (W3) sample, which peaked below 3000 OTUs (Figure S1).

**Figure 4:**
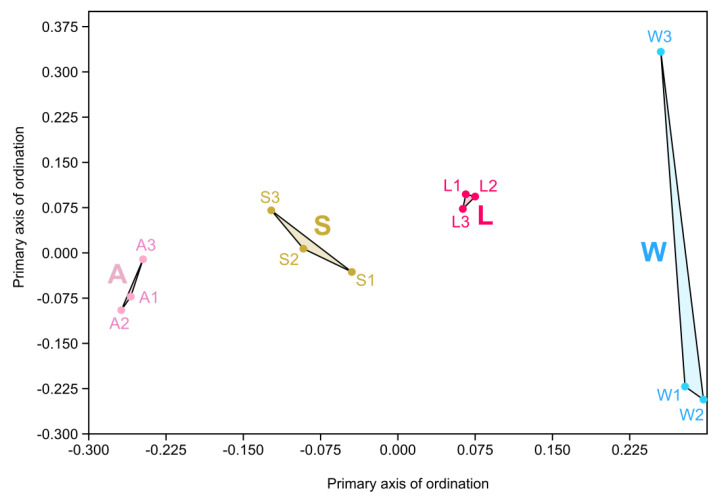
Non-metrical multidimensional scaling (NMDS) plot of the Bray–Curtis dissimilarity matrix of the OTU distribution in *P. cyanorosea* adults (pink), larvae (red), ambient seawater (blue) and sediment (ochre). Ordination stress was 0.12 in the scale of 0–1.0. Abbreviations: A: adults; L: larvae; W: seawater; S: sediment.

Following Bray–Curtis based NMDS analysis, the clustering of each sample within its respective group along the horizontal axis was evident. Notably, the larvae cluster occupied a central position in the multidimensional analyses, being more aligned with the environmental sample clusters (seawater and sediment) in relation to their respective parental sponge cluster (Figure 4). Statistically significant differences in community structure (PERMANOVA) were identified between the symbiont microbial communities from the different sponge life cycles and the environmental samples (F = 6.558, *p* = 0.001), with 71% variation explained by the sample group (R^2^ = 0.71). Dispersion analyses (PERMDISP) corroborated the results from the multivariate analyses results (*p* = 0.847).

### 3.4. OTU-Level and Core Microbiota Analyses

By selecting the OTUs that were unique to a particular sample, sample groups or combinations between these groups, we confirmed that very few of the seawater microbiota (0.15%) were not present in any of the other groups. In particular, a set of 15 OTUs were unique to the seawater (total of reads in seawater samples = 9983), 173 OTUs (1.66%) were unique to the larvae (total of reads in larvae samples = 10,368), 583 OTUs (5.43%) were unique to the adults (total of reads in adult samples = 10,732), and 1483 OTUs (5.79%) were unique to the sediment samples (total of reads in sediment samples = 25,605) (Table S2). Only five OTUs were uniquely present in both the adult and larvae samples (unclassified Gammaproteobacteria, *Ferrimonas*, two assigned as unclassified Alphaproteobacteria and unclassified Vibrionaceae) and two OTUs were uniquely found in both seawater and sediment samples (*Lentilitoribacter* and unclassified Rhodobacteriaceae). Sponge life stages and seawater shared only one unique OTU (*Psychrosphaera*) while sharing three unique OTUs (unclassified Enterobacteriaceae, unclassified *Nitrosopumilus* and *Halomonas*) with the sediment samples. Meanwhile, the larvae, seawater and sediment samples shared two unique OTUs (both classified as being from the *Bacteroides* genus) and the adult samples did share any common unique OTU with the environmental samples. Details on the taxonomic classification of these unique and uniquely shared OTUs are presented in Table S4. Neither of these unique and uniquely shared OTUs was amongst the 30 dominant OTUs and had relative abundance inferior to 0.2% (Table S4).

Unique patterns in abundant and prevalent OTUs were evident when examining each sample group. Even though being also present at an abundance >1.0% in the other samples, the unclassified Alphaproteobacteria OTUs were significantly enriched in the parental sponges. As previously mentioned, adults were the reservoir for archaea amongst the samples, particularly from the Thaumarchaeota phylum. Nearly 99% of these thaumarchaeal OTUs were from the genus *Nitrosopumilus*. The Alteromonodales order was found at a mean relative abundance of 24% in all larvae samples, in particular OTUs corresponding to the *Pseudoalteromonas* genus; with this bacterial genus also being a major member (41% of abundance) of the bacterial communities in one of the seawater sample (W3). On the other hand, *Arcobacter* covered one-third of the microbial composition of the other ambient seawater. Following unclassified Bacteria, unclassified Proteobacteria and unclassified Gammaproteobacteria OTUs, sediment samples were also dominated by OTUs classified as either unclassified Bacteroidetes and unclassified Planctomycetaceae.

**Figure 5:**
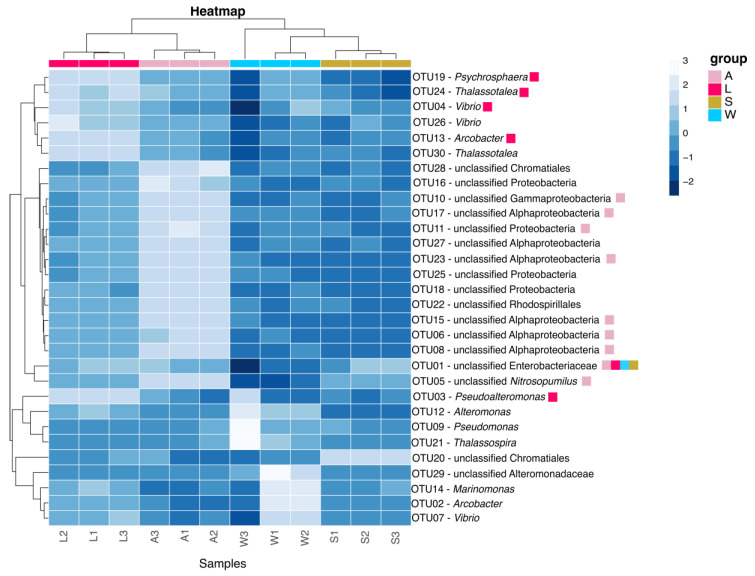
Heatmap with the top 30 most abundant OTUs detected in the different life stages of the marine sponge *P. cyanorosea*, ambient seawater and sediment. For plotting purposes, Z-transformation was applied for the logarithmic conversion of the total read count for each top OTU. The OTUs comprising the strict core microbiota for all samples, adults and larvae are highlighted as squares colored according to the legend (upper left). A: adults; L: larvae; W: seawater; S: sediment.

The top 30 OTUs corresponded to approximately 70% of the total microbial composition encountered in all the samples (Figure 5; Table S5). Apart from the 2nd and the 13th most abundant OTUs (*Arcobacter*), the remainder were from the Alpha- (unclassified Alphaproteobacteria, *Thalassospira*), Gamma- (unclassified Enterobacteriaceae, *Alteromonas*, unclassified Alteromonadaceae, *Pseudoalteromonas*, *Psychrosphaera*, *Thalassotalea*, *Marinomonas*, unclassified Vibrionaceae, *Vibrio*) and Epsilonproteobacteria (*Arcobacter*) classes. The most abundant OTU, identified as unclassified Enterobacteriaceae, totaled 16% of the microbial communities that were common in all samples.

Moreover, 14 out of these top30 abundant OTUs (OTU1, OTU3, OTU5, OTU7, OTU12, OTU13, OTU14, OTU17, OTU19, OTU20, OTU21, OTU24, OTU26; Table S4) were 100% identical to OTUs from the curated reference sponge microbiome database. In the case of the 13 unique OTUs and uniquely shared OTUs between groups, only two OTUs (OTU1045 was shared by sponge life stages and seawater and OTU52 was shared between larvae and environmental samples; Table S5) were 100% identical to OTUs from the curated reference sponge microbiome database. The significantly enriched OTUs were present in about 5.0% of the samples in the SpongeEMP database, including significant hits for 60 sponge hosts (Table S6).

The unclassified Enterobacteriaceae OTU was the dominant member of the core microbiota shared by all samples at a minimum abundance of 0.01% (Figure S2). By respecting this detection threshold, but now assuming a 100% prevalence (considering only to the OTUs found in all samples), it was possible to determine strictly the core microbiota of each group that was analyzed. Essentially, the adult core microbiota was composed of nine OTUs: unclassified Enterobacteriaceae (OTU01), unclassified *Nitrosopumilus* (OTU05), unclassified Gammaproteobacteria (OTU10), unclassified Proteobacteria (OTU11), and 3 OTUs classified as unclassified Alphaproteobacteria (OTU06, OTU08, OTU15, OTU17 and OTU23). Six OTUs represented the larval core microbiota, including unclassified Enterobacteriaceae (OTU01) with the other five belonging to the genera *Pseudoalteromonas* (OTU03), *Vibrio* (OTU04), *Arcobacter* (OTU13), *Psychrosphaera* (OTU19) and *Thalassotalea* (OTU24). By performing a cross-section analyses of the core microbiota between each sponge life stage (adults and larvae) group with both environmental samples (ambient seawater and sediment), it was clear that the top OTU, unclassified Enterobacteriaceae (OTU01), remained as the exclusively shared core OTU in all samples at a 0.01% abundance threshold. This was also verified when assessing the core microbiota of each environmental sample isolated versus each sponge life cycle (adults vs. seawater, adults vs. sediment, larvae vs. sediment). The adult sponge and its respective offspring also shared the top unclassified Enterobacteriaceae OTU as the major member of its core microbiota. Interestingly, the core microbiota of one adult-larvae pair, A3-L3, was composed of three OTUs: unclassified Enterobacteriaceae, *Pseudoalteromonas* (OTU03) and *Vibrio* (OTU04). Under the assumed conditions (minimum relative abundance of 0.01% and prevalence of 100%), no strict core microbiota was observed between the seawater and sediment samples.

### 3.5. Global Analyses of Homoscleromorpha-Associated Prokaryotic Microbiota

By including the 16S rRNA gene sequencing data of 88 Homoscleromorph sponges to the 12 samples considered in our survey, a sum of 18,459,306 sequence reads was assembled into 84,356 OTUs. A considerable variation for the alpha diversity metrics was observed (Table S7), given that a different number of samples were available at the SMP database for each Homoscleromorph genus/species (e.g., there are 43 samples for *Plakortis* spp. while just one single sample for *Plakinastrella* spp.). Six larvae samples from *Oscarella lobularis*, one from *Corticium candelabrum* and two *Plakina trilopha* specimens showed a low number of OTUs (<100) when compared to the other sponges. The highest number of OTUs was detected in one larvae sample from *C. candelabrum* (11,240), followed by the sediment and *Plakortis* samples. Indeed, this plakinid species, particularly the species *Plakortis angulospiculatus* and *Plakortis halichondrioides*, harbored the most diverse microbial assemblages, followed by *Pseudocorticium*, *Corticium*, *Plakina* and *Oscarella*.

The structure of Homoscleromorpha-associated microbial communities (Figure S3) revealed a clear and well-arranged clustering of *Plakortis*, *Pseudocorticium jarrei* and all the *C. candelabrum* specimens (except for one sample, what can be explained by its lower richness), with all of them closely associated or even overlapping with each other. In its turn, the symbiont communities of *Oscarella* and *Plakina trilopha* varied significantly between the samples, which can be visualized by their wide scattered pattern in the NMDS plot, in particular for some *Oscarella* larvae samples. The microbiota of *P. cyanorosea* aggregated relatively isolated from these other Homoscleromorph genera. In accordance with the former observation, the *P. cyanorosea* larvae cluster was to a large extent positioned closer to the sediment and ambient seawater in relation to their respective parental cluster. Statistical differences between the sponge species were detected by PERMANOVA (F = 5.53; *p* = 0.003) and the microbiota were found to differ significantly between the samples (41%), as determined by PERMDISP analyses (*p* = 1.05 × 10^−11^), which means that variances were heterogenous.

Following the large proportion of microbial communities found in unclassified Bacteria (Figure S4), the Alpha- and Gamma-proteobacteria classes dominated the composition of the Homoscleromorpha microbiota at the class level, with the alphaproteobacterial symbionts more enriched in the *C. candelabrum*, *O. lobularis* and the *P. cyanorosea* parental samples when compared to other Plakinidiae species and *P. cyanorosea* larvae (Figure S5). The Enterobacteriaceae family constituted up to 80% of the microbial communities in some of the *O. lobularis* larvae samples, while the alphaproteobacterial family Rhodobacteraceae was prominent in most of the adults in this Oscarellidae species (Figure S6). Three *Oscarella* adults had a Verrucomicrobia abundance of around 7.0–8.0% in relative abundance, while the phylum Poribacteria was more commonly detected in the Plakinidae samples, even though at a low relative abundance (around 1.0 to 3.0% on average). Thaumarchaeota was the dominant archaeal phylum in all Homoscleromorpha sponges (Figure S4). As observed in the *P. cyanorosea* adults, the majority of these archaeal symbionts were classified as *Nitrosopumilus*.

## 4. Discussion

We report here for the first time the characterization of bacterial and archaeal communities associated with the marine sponge *P. cyanorosea* through a 16S rRNA metabarcoding approach. The microbial communities found in the three sampled adult individuals were richer and less diverse than those detected in their respective offspring. Higher richness and diversity indexes were encountered for the sediment samples, while lower alpha-diversity metrics were determined for the surrounding seawater samples. This was also reported in the first global survey of the sponge microbiome, in which both the ambient surrounding seawater and sediment were also assessed [66]. Statistic differences for all the alpha-diversity metrics were observed between adults and larvae, between sponges (adults and larvae) and environmental samples (seawater and sediment) and between all the sample groups, with sediment microbiota being significantly different in relation to the other groups (Figure 3; Table S3). The larvae microbiota has previously been reported to be less rich and less diverse than the microbial communities living in their respective parents for some demosponge species, such as *Amphimedon queenslandica* [27], and *Clathria prolifera* and *Halichondria bowerbani* [19]; however, the opposite has also been reported, for instance in the Great Barrier Reef sponge *Rhopaloeides odorabile* [16]. The higher microbial diversity here observed in the *P. cyanorosea* offspring may result from the deposition of environmental microorganisms on the surface of the non-feeding larvae [24], in particular during their later stages in sponge development, just before the release into the seawater environment.

Following beta-diversity analyses, the larvae microbial communities clearly clustered between the sediment and the seawater microbiota. The adult cluster was positioned closer to the sediment cluster than the larvae cluster in the Bray–Curtis-based NMDS plot (Figure 4). Thus, the closer proximity of the adult cluster to the sediment cluster rather than the larvae cluster hints that environmental acquisition is the main transmission mode in *P. cyanorosea*. Indeed, both of the novel *Plakina* species previously isolated from this shallow tide pool (*P. cyanorosea* and *P. cabofriense*) were found on the under-surfaces of rocks and were covered by the surrounding sediment [45]. This could be a strategy employed by the sponge to help it establish in shadowed or semi-dark microenvironments; an ecological trait that is widely distributed in Homoscleromorpha [35]. However, to what extent the sediment does, in fact, affect the composition of the microbiota in these plakinid sponges in comparison with the seawater can only be resolved with further research in this area.

Community-composition level analyses highlighted the dominance of the alpha- and gammaproteobacterial communities in the adults, with most of these being OTUs which ranked in an unresolved status (unclassified Alpha- and unclassified Gammaproteobacteria). The Thaumarchaeota phylum, specifically from the *Nitrosopumilus* genus, accounted for approximately ten percent of the microbial composition of *P. cyanorosea*. The results for the adult microbiota are in agreement with what has been previously reported in the overall prokaryotic microbial composition of marine sponges [62]. It is noteworthy that Thaumarchaeota has also been detected in relative abundances that are on average greater than 5.0% in the other Homoscleromorpha sponges which were evaluated in our global analyses. Indeed, the prevalence of this archaeon phylum compares favorably with what has been recently reported in deep-sea hexactinellids and demosponges [69]: the predominance of *Nitroposumilus* symbionts appears to be correlated to the individual sponges and does not strictly follow a species-specific trait. The functional roles being undertaken by these thaumarchaeons, mainly involving the detoxification of ammonia and nitrogenated byproducts for the sponge host, [70,71] are likely to be widely conserved in all sponge classes, and, therefore, constituting a key component of their core microbiome. VT of these archaeal symbionts could also be taking place in *P. cyanorosea* despite their lower abundance in the sampled larvae (around 1.0% on average). VT of Thaumarcheota has been confirmed by complementary fluorescence microscopy in microbial diversity surveys for other sponge species [72,73]. Future use of specific fluorescence probes for these thaumarchaeal symbionts in larvae and embryos followed by complete microscopic analyses would however be required to support the hypothesis of their VT in *P. cyanorosea*. However, environmental capture cannot be ruled out, given that a recent report on non-Homoscleromorpha tropical sponges has reported that four core archaeal OTUs were closely related to environmental clone sequences [74].

Other significant shifts were observed in the prokaryotic composition when comparing the adult to larvae microbial populations. A depletion in the abundance of Alphaproteobacteria was replaced by an increase of the Gammaproteobacteria class, in particular chemoorganotrophic representatives from the Enterobacteriaceae, Vibrionaceae and Alteromonodaceae families, with the latter two not being present in higher abundance in adults. The specific enrichment in larvae of the Vibrionaceae and Alteromonodaceae families could be as a result of their origin from the seawater since both families are composed by ubiquitous marine bacterial genera (including *Vibrio*, *Pseudoalteromonas*, *Psychrosphaera, Thalassotalea*) and were also found in higher abundance in the seawater samples. In our global analyses, the higher abundance of *Pseudoalteromonas* appeared to be unique to the larvae of *P. cyanorosea* when compared to other Homoscleromorpha specimens. Nevertheless, cultivable strains with specialized genomic content encoding for properties that may be involved in a symbiotic lifestyle have previously been reported from both the *Vibrio* [75] and *Pseudoalteromonas* [76,77] strains isolated from sponges. Therefore, a possible long-term association with the sponge host and the likelihood of maternal transmission also occurring be running in *P. cyanorosea* cannot be completely discarded for these gammaproteobacterial genera.

Together with members of the Clostridia and Bacteroides classes, whose relative abundances were also higher in the larvae microbiota, these gammaproteobacterial families are known to be prolific degraders of complex high-molecular-weight (HMW) polymeric substrates [78,79]. The deposition of seawater microbes on the surface of larvae during their dispersal has already been reported to occur in sponges transiting between VT and HT modes [24]. Hence, we suggest that the presence of these heterotrophic partners, transient or not, could be strategic for the sponge host during the larvae settlement and metamorphosis into the juvenile form. The simpler monomeric nutrients released by the hydrolytic action of these chemoheterotrophic prokaryotes may energetically aid the sponge until its aquifer system is completely developed and the filter-feeding activity can begin to function properly, as has been previously suggested [27]. Additionally, bacterial isolates from the *Paraclostridium* genus (Clostridia) have been isolated from *Plakina* species (*P. cyanorosea* and *P. cabofriense*) that are endemic in this tidal pool [47] and, therefore, although their low abundance in adults, they were found to be present in culture-based surveys.

The abundance of the Epsilonproteobacteria class, represented mainly by the *Arcobacter* OTUs, was also slightly elevated in the larvae (4.0–5.0% of relative abundance), again reflecting the potential impact of seawater on the offspring microbiota. This Campylobacteraceae genus dominated two seawater samples but levels were much lower in the third sample (W3). This may be as a result of the sampling location, which was a little more distant to the seashore when compared to the other two seawater samples and is likely to be more susceptible to the vigorous “washing effect” of the tidal movement. The W3 sample was taken from seawater surrounding the third adult sponge (A3), which was amongst the sponges collected far from the shore and also had a bacterial community composition which differed slightly from the other two adult sponge samples. Given that this tidal pool is frequently used as a recreational area in low-tide conditions and has close proximity to one of the major touristic beaches of this coastal area, it is likely that the anthropogenic impact would be reflected in the local seawater microbial community. The dominance of *Arcobacter* denotes a strong indication of human activity in this tidal pool. This genus includes several food-borne and opportunistic enteropathogenic species of humans and animals [80], whose presence in marine environments has been associated with human pollution and risk infection [81,82,83,84]. As a consequence of its zoonotic potential, *Arcobacter* has also been reported as a member of the microbiota in the larvae of other invertebrates, harboring key genomic features related to resistance to environmental conditions [85]. It is therefore likely that the *P. cyanorosea* larvae probably acquired this OTU from the seawater surroundings.

For the unclassified Enterobacteriaceae OTU, which dominated the adult and larvae microbiota and was the main member of the sponge core microbiota, both VT and HT could potentially be operating. This could be taking place either by a dual mixed model of transmission or by an indirect VT mechanism, where environmental symbionts actively acquired by the parents are eventually integrated during the embryonic/larvae development [20]. Interestingly, Enterobacteriaceae was not encountered at higher abundance in the seawater samples (around 1.0–2.0% of relative abundance) in stark contrast with both the *P. cyanorosea* life stages. This is similar to a recent study on the different developmental stages of a *Tedania* sponge sampled from a Chinese Bay [18]. The relative abundance of this Enterobacterales family was also markedly higher in *Tedania* adults when compared to the surrounding water. In addition, the Enterobacteriaceae family constituted up to one-fourth of the microbial communities in the *Tedania* larvae and reached levels as high as almost half of the microbiota composition detected in an immediate post-larva stage [18]. Furthermore, Enterobacterales/Enterobacteriaceae have also been found at quite high levels, or indeed as amongst the dominant OTUs in marine demosponges, such as *Haliclona fulva* [86] and *Mycale* spp. [87]. While it appears likely that environmental acquisition was involved in the selective enrichment of Enterobacteriaceae in *P. cyanorosea* adults and their apparent passage to the offspring, the exact mechanisms underlying this will require further investigation to support this apparent phenomenon.

Nearly half of the top 30 most abundant OTUs had hits at complete identity (100%) with the deblurred OTUs from the curated reference sponge microbiome database. Nonetheless, a low percentage of these closest OTUs relatives were significantly enriched in the host specimens from the Sponge EMP project. There is a number of possible explanations for this result. Firstly, the dominant microbiota in *P. cyanorosea* is simply not enriched in the sponge specimens in the Sponge EMP project. Thus, the surrounding environment would be the original source of this overall microbiota, which is exemplified by the seawater OTUs being encountered in other samples, in agreement with the surrounding seawater constituting a seedbank for the assimilation of horizontally-transmitted or even “sponge-specific microbial clusters” [16]. Secondly, this low percentage of “sponge-enriched microbial clusters” could be related to intrinsic features of the SMP database. In its conception, three databases (SILVA, Greengenes and RDP) were incorporated for taxonomic classification of the OTUs [62], considering the confirmed impact on the community composition deriving from the choice of one sole taxonomic database in microbiome-based studies [88,89]. We adopted the latest release of the RDP database for the taxonomic assignment of the study samples and, congruently, the reference curated sponge microbiome database created with the original SMP data. This could have led to a low percentage of significantly enriched subOTUs in the host samples belonging to the Sponge EMP server. Finally, and importantly, the Sponge EMP database has a low number of Homoscleromorpha specimens, particularly from the *Plakina* genus. In consequence, the typical microbiota composition of these sponges might be not well-covered in the most recent version of the database, lacking the necessary data for OTUs that might be significantly-enriched in this sponge class, which seems to be unlike what has been reported in the other classes [25,90].

In this context, we compared the samples from our survey with the 16S rRNA gene sequencing data from Homoscleromorpha specimens present in the SMP database. Consequently, it became clear that the *P. cyanorosea* microbiota possesses a lower to a middle microbial richness on a scale ranging from *Oscarella* to *Plakortis*. Shannon’s diversity index values matched with those observed for *Plakina trilopha* but were not higher as those encountered for *Plakortis* hosts. The Homoscleromorpha microbial structure provides interesting insights about these sponges as well. The community of *Corticium*, *Pseudocorticium* and *Plakortis*, for which more samples were available in the SMP database, are similar to each other, with more conserved microbial taxa. On the other hand, the structure of the *Oscarella* and *Plakina* microbiomes range considerably as revealed by their scattered distribution in the NMDS plot. Larvae samples are only available for *Corticium* and *Oscarella*: for the first, larvae and adult microbial communities are aggregated together, which somehow agrees with the VT evidence already confirmed for *C. candelabrum* [72,91,92]. In contrast, the microbiota of the *Oscarella* larvae differed greatly from its respective parents, which are most similar to each other in comparison with their larvae. Some *Oscarella* larvae samples are solely dominated by two OTUs or even enriched in Enterobacteriaceae, while the adult microbial community composition exhibits an apparent more resilient sponge microbiota, harboring a high abundance of alphaproteobacterial symbionts (Rhodobacteraceae), which have previously been reported in these sponges [93]. Further work will be required to determine whether HT is the major mechanism responsible for these microbiota patterns in various *Oscarella* developmental stages, as it seems to be in *P. cyanorosea*.

Thus, the data presented here together with previous reports, appear to indicate that *P. cyanorosea* is a low microbial abundance (LMA) sponge, a status already ascribed to the sponge based on involving electron microscopy observations in its original taxonomic description [45]. It has been suggested that this LMA sponge could be associated with the extreme and ever-changing conditions of the tidal pool ecosystem which is home to the sponge and may affect a long-term stability of the sponge’s microbiome [45]. In contrast, the New Caledonian *P. kanaky* sponge species, which was microscopically classified as a high microbial abundance (HMA) sponge [41], is found in submarine caves typically located on the outer slopes of coral reefs, and would therefore typically not be exposed to anthropogenic interference such as *P. cyanorosea* likely encounter in the tidal pool where it is found. In this HMA-LMA dichotomy, Oscarellidae are postulated to be LMA sponges and Plakinidae as HMA sponges [49]. However, it should be noted that, while at first glance the *Plakina* genus appears to be really dominated by HMA species, some LMA *Plakina* species have also recently been reported [38]. Thus, the HMA-LMA dichotomy for Homoscleromorpha hosts may not be as straight forward as originally thought. Together with the host species, signature microbial taxa [94] and essential core microbial functions [95], habitat is also a key factor in determining the microbial abundance in the sponge holobiont. An increase of microbiome-based assessments on this sponge class will reveal which Homoscleromorpha family are HMA or LMA species, if such a trend exists. Additionally, it is clear that the HMA–LMA dichotomy needs to be revisited [96]—to avoid generalizations based either exclusively on the cytological description of prokaryote morphotypes in the sponge mesohyl or the presence of diagnostic microbial taxa. The abundance of surrounding seawater samples and the sponge morphology should also be considered for this particular classification [97,98,99].

Overall, LMA hosts appear to have evolved to possess less dense tissues and more developed aquifer systems, which allow them to efficiently pump particulate organic matter (POM) from higher seawater volumes [99,100]. The *P. cyanorosea* anatomy possesses these features [45] and perhaps provides some basis for the enrichment in the offspring of chemoorganotrophic microbial members, which are active degraders/colonizers of POM [101,102]. Coupled with the low phylum-level diversity and the less rich microbial community structures compared to their HMA counterparts, *P. cyanorosea* possesses a certain dominance of environmentally-acquired microbial taxa, matching with traits broadly described in other LMA hosts [103,104,105,106]. The presence of environmental microorganisms appears to increase during the transition to the larvae phase in *P. cyanorosea*. HT has previously been considered as a crucial contributor to microbial community diversity in LMA sponges, allowing them to quickly respond to environmental stresses by selectively incorporating local and functionally advantageous microbial phenotypes [107,108]. Our results suggest that *P. cyanorosea* could be such an example of an LMA sponge, which has adapted to promptly capture the environmental microorganisms present in its immediate ecosystem to survive in the inconstant tidal pool habitat. However, maternal passage of symbionts, including the top enterobacterial OTU and the thaumarchaeal *Nitrosopumilus*, may also be occurring. The role of HT in the transmission of the sponge microbiome has been reported in recent studies [17,20]. Soon a mixed model will emerge as the better alternative for the sponge holobiont. In this model, both transmission modes contribute in varying degrees and explain their role in a wide array of different sponge hosts inhabiting very distinctive environments.

## 5. Conclusions

The microbiology of Homoscleromorpha is in its infancy. The present study set out to determine the diversity and composition of the bacterial and archaeal communities associated with adult and larvae stages of the marine sponge *P. cyanorosea*, a novel species of the *Plakina* genus just described in a shallow-water ecosystem in the Brazilian Southeast coast. Richness and diversity indexes were closer but significantly different for adults and larvae. The microbiota composition in the latter was more structurally related to the ambient seawater and sediment, with respect to their respective parents. The Gamma- and Alpha-proteobacteria classes, together with the thaumarcheon *Nitrosopumilus*, were predominant in the parental specimens, while the offspring microbial assemblages were prominent in typical chemoheterotrophic bacterial taxa, including the orders Enterobacterales, Alteromonodales, Vibrionales (Gammaproteobacteria), Bacteroidales (Bacteroidetes) and Clostridiales (Firmicutes). Microbial indicators of anthropogenic interference are evident in the tidal pool seawater. An enterobacterial OTU was the main member of the strict core microbiota found across all sample groups and between both sponge life stages. A low share of the dominant OTUs encountered in all samples and unique OTUs encountered in the *P. cyanorosea* life stages were present in hosts specimens included in the current version of the Sponge Microbiome Project. Amongst the Homoscleromorpha microbiome continuum, *P. cyanorosea* seems to be located in a central position in alpha diversity terms, while not sharing similar microbial communities to other well-sampled genera, such as *Corticium*, *Pseudocorticium* and *Plakortis*. Lastly, *P. cyanorosea* is an LMA sponge, which may take advantage of active horizontal transmission as a means of survival and maintenance of a minimal functionally stable microbiota to help it manage the ever-changing and unpredictable conditions in its native habitat. Without ruling out a minor contribution by vertical transmission, our findings suggest that the acquisition of microorganisms directly from ambient sources appears to markedly influence the microbiota of *P. cyanorosea*, particularly in its transition from adult to larvae.

## Supplementary Materials

The following are available online at https://www.mdpi.com/2076-2607/8/12/1978/s1, Figure S1: Rarefaction curves of 16S rRNA sequence diversity in sponge and environmental samples. The total OTU number is depicted as a function of the number of sequences derived from Illumina sequencing. Lines are colored in accordance with each sample group, as follow: pink for adults, red for larvae, blue for seawater and ochre for sediment, Figure S2: Heatmap of the core microbiota with the increasing detection thresholds showed on the *x* axis, Figure S3: NMDS plot of the Bray-Curtis dissimilarity matrix based on OTU distribution of Homoscleromorpha sponges (88 hosts from the SMP database plus six samples of *P. cyanorosea* from the current work) and environmental samples (seawater and sediment) selected for the global survey. Ordination stress was 0.16 in the scale 0–1.0. A: adults; L: larvae; W: seawater; S: sediment; Figure S4: Phylum-level relative abundance of the community prokaryotic composition of the Homoscleromorpha sponges and environmental samples. A: adults; L: larvae; W: seawater; S: sediment; Coca_A: *Corticium candelabrum* (adults); Coca_L: *Corticium candelabrum* (larvae); Oslo_A: *Oscarella lobularis* (adults); Oslo_L: *Oscarella lobularis* (larvae); Pl_A: *Plakortis* sp. (adults); Plan_A: *Plakortis angulospiculatus* (adults); Plha_A: *Plakortis halichondrioides* (adults); Plakinastrella_A: *Plakinastrella* sp.; Pltr_A: *Plakina trilopha* (adults); Psja_A: *Pseudocorticium jarrei* (adults), Plsi_A: *Plakortis simplex* (adults), Figure S5: Class-level relative abundance of the community prokaryotic composition of the Homoscleromorpha sponges and environmental samples. A: adults; L: larvae; W: seawater; S: sediment; Coca_A: *Corticium candelabrum* (adults); Coca_L: *Corticium candelabrum* (larvae); Oslo_A: *Oscarella lobularis* (adults); Oslo_L: *Oscarella lobularis* (larvae); Pl_A: *Plakortis* sp. (adults); Plan_A: *Plakortis angulospiculatus* (adults); Plha_A: *Plakortis halichondrioides* (adults); Plakinastrella_A: *Plakinastrella* sp.; Pltr_A: *Plakina trilopha* (adults); Psja_A: *Pseudocorticium jarrei* (adults), Plsi_A: *Plakortis simplex* (adults),*order, Figure S6: Family-level relative abundance of the community prokaryotic composition of the Homoscleromorpha sponges and environmental samples. A: adults; L: larvae; W: seawater; S: sediment; Coca_A: *Corticium candelabrum* (adults); Coca_L: *Corticium candelabrum* (larvae); Oslo_A: *Oscarella lobularis* (adults); Oslo_L: *Oscarella lobularis* (larvae); Pl_A: *Plakortis* sp. (adults); Plan_A: *Plakortis angulospiculatus* (adults); Plha_A: *Plakortis halichondrioides* (adults); Plakinastrella_A: *Plakinastrella* sp.; Pltr_A: *Plakina trilopha* (adults); Psja_A: *Pseudocorticium jarrei* (adults), Plsi_A: *Plakortis simplex* (adults), Table S1: List of sponge hosts selected from the Sponge Microbiome Project (SMP) and used for the global analyses of the Homoscleromorpha-associated microbiota, Table S2: Sequence statistics and alpha diversity metrics of sponge-associated prokaryotic communities found in different life stages of *P. cyanorosea*, ambient seawater and sediment, Table S3: Statistical assessment of the alpha diversity metrics for the pairwise comparisons between two groups (sponge vs. environment; adults vs. larvae) by the *t*-tests and all groups by One-Way ANOVA, Table S4: Taxonomic classification of unique and uniquely shared OTUs found in each sample group or between combinations of sample groups, Table S5: Taxonomic classification and relative abundance of the top30 OTUs detected in the different life stages of *P. cyanorosea* and environmental samples, Table S6: List of sponge hosts containing the significantly enriched closest subOTUs relatives of the top30 and 13 unique and uniquely shared OTUs found in *P. cyanorosea* and environmental samples, Table S7: Alpha diversity metrics obtained for the Homoscleromorph sponges and environmental samples in the global analyses.
