# Supplementary material for: Not That Close to Mommy: Horizontal Transmission Seeds the Microbiome Associated with the Marine Sponge Plakina cyanorosea
Source: Microorganisms. 2020 Dec 12;8(12):1978. doi: 10.3390/microorganisms8121978 (PMC7764410; doi:10.3390/microorganisms8121978)
Supplement: Supplementary file 1 [file microorganisms-08-01978-s001.zip › microorganisms-954541-suppl-Table.docx]

Article

Not that Close to Mommy: Horizontal Transmission Seeds the Microbiome Associated with the Marine Sponge *Plakina cyanorosea*

Bruno F. R. Oliveira ^1,2^, Isabelle R. Lopes ^1^, Anna L. B. Canellas ^1^, Guilherme Muricy ^3^,
Alan D.W. Dobson ^2,4^ and Marinella S. Laport ^1,^*

^1^ Laboratório de Bacteriologia Molecular e Marinha, Instituto de Microbiologia Paulo de Góes, Universidade Federal do Rio de Janeiro, 21941902 Rio de Janeiro, Brazil; bfroliveira@micro.ufrj.br (B.F.R.O.); rodrigueslopes.isabelle@gmail.com (I.R.L.); annaluizabcc@gmail.com (A.L.B.C.)

^2^ School of Microbiology, University College Cork, T12 Y960 Cork, Ireland; a.dobson@ucc.ie

^3^ Laboratório de Biologia de Porifera, Museu Nacional, Universidade Federal do Rio de Janeiro, 20940040 Rio de Janeiro, Brazil; muricy@mn.ufrj.br

^4^ Environmental Research Institute, University College Cork, T23 XE10 Cork, Ireland

***** Correspondence: marinella@micro.ufrj.br

Received: 18 September 2020; Accepted: 25 November 2020; Published: date

**Table S1.** List of sponge hosts selected from the Sponge Microbiome Project (SMP) and used for the global analyses of the Homoscleromorpha-associated microbiota.

| **Genus/Species** | **Family** | **Life stage** | **Attributed ID Code** | **SRA Accession Number** | **Qiita ID** |
| --- | --- | --- | --- | --- | --- |
| *Oscarella lobularis* | *Oscarillidae* | Adult | Oslo_A_1 | ERX1845334 | 2365:10793.10533.Olo.14.1a |
| *Oscarella lobularis* | *Oscarillidae* | Adult | Oslo_A_2 | ERX1845335 | 2365:10793.10533.Olo.14.1b |
| *Oscarella lobularis* | *Oscarillidae* | Adult | Oslo_A_3 | ERX1845336 | 2365:10793.10533.Olo.14.2a |
| *Oscarella lobularis* | *Oscarillidae* | Adult | Oslo_A_4 | ERX1845337 | 2365:10793.10533.Olo.14.3a |
| *Oscarella lobularis* | *Oscarillidae* | Adult | Oslo_A_5 | ERX1845338 | 2365:10793.10533.Olo.14.4a |
| *Oscarella lobularis* | *Oscarillidae* | Adult | Oslo_A_6 | ERX1845339 | 2365:10793.10533.Olo.14.5a |
| *Oscarella lobularis* | *Oscarillidae* | Adult | Oslo_A_7 | ERX1842854 | 2334:10793.10533.EMP.GR..6.B |
| *Oscarella lobularis* | *Oscarillidae* | Adult | Oslo_A_8 | ERX1842853 | 2334:10793.10533.EMP.GR..6.A |
| *Oscarella lobularis* | *Oscarillidae* | Larvae | Oslo_L_1 | ERX1845340 | 2365:10793.10533.OslL.1.1a |
| *Oscarella lobularis* | *Oscarillidae* | Larvae | Oslo_L_2 | ERX1845341 | 2365:10793.10533.OslL.1.1b |
| *Oscarella lobularis* | *Oscarillidae* | Larvae | Oslo_L_3 | ERX1845342 | 2365:10793.10533.OslL.2.1a |
| *Oscarella lobularis* | *Oscarillidae* | Larvae | Oslo_L_4 | ERX1845343 | 2365:10793.10533.OslL.2.1b |
| *Oscarella lobularis* | *Oscarillidae* | Larvae | Oslo_L_5 | ERX1845344 | 2365:10793.10533.OslL.3.1a |
| *Oscarella lobularis* | *Oscarillidae* | Larvae | Oslo_L_6 | ERX1845345 | 2365:10793.10533.OslL.3.1b |
| *Oscarella lobularis* | *Oscarillidae* | Larvae | Oslo_L_7 | ERX1845346 | 2365:10793.10533.OslL.3.2b |
| *Oscarella lobularis* | *Oscarillidae* | Larvae | Oslo_L_8 | ERX1845347 | 2365:10793.10533.OslL.3.3a |
| *Oscarella lobularis* | *Oscarillidae* | Larvae | Oslo_L_9 | ERX1845347 | 2365:10793.10533.OslL.3.3b |
| *Pseudocorticium jarrei* | *Oscarillidae* | Adult | Psja_A_1 | ERX1233018 | 1257:10346.SS.29.1 |
| *Pseudocorticium jarrei* | *Oscarillidae* | Adult | Psja_A_2 | ERX1233019 | 1257:10346.SS.29.2 |
| *Pseudocorticium jarrei* | *Oscarillidae* | Adult | Psja_A_3 | ERX1233020 | 1257:10346.SS.29.3 |
| *Pseudocorticium jarrei* | *Oscarillidae* | Adult | Psja_A_4 | ERX1839853 | 2370:10793.SS.29.1.1181936 |
| *Pseudocorticium jarrei* | *Oscarillidae* | Adult | Psja_A_5 | ERX1839854 | 2370:10793.SS.29.2.1182178 |
| *Pseudocorticium jarrei* | *Oscarillidae* | Adult | Psja_A_6 | ERX1839855 | 2370:10793.SS.29.3.1181799 |
| *Corticium candelabrum* | *Plakinidae* | Not provided (Adult) | Coca_A_1 | ERX1842824 | 2334:10793.10533.Coca2 |
| *Corticium candelabrum* | *Plakinidae* | Not provided (Adult) | Coca_A_2 | ERX1842825 | 2334:10793.10533.Coca3 |
| *Corticium candelabrum* | *Plakinidae* | Not provided (Adult) | Coca_A_3 | ERX1842826 | 2334:10793.10533.Coca4 |
| *Corticium candelabrum* | *Plakinidae* | Larvae | Coca_L_1 | ERX1845156 | 2365:10793.10533.Cca.3.1a |
| *Corticium candelabrum* | *Plakinidae* | Larvae | Coca_L_2 | ERX1845157 | 2365:10793.10533.Cca.3.1b |
| *Corticium candelabrum* | *Plakinidae* | Larvae | Coca_L_3 | ERX1845158 | 2365:10793.10533.Cca.3.2a |
| *Corticium candelabrum* | *Plakinidae* | Larvae | Coca_L_4 | ERX1845159 | 2365:10793.10533.Cca.3.3a |
| *Corticium* sp. | *Plakinidae* | Larvae | Coca_L_5 | ERX1845160 | 2365:10793.10533.Cca.3.4a |
| *Corticium* sp. | *Plakinidae* | Larvae | Coca_L_6 | ERX1845161 | 2365:10793.10533.Cca.3.5a |
| *Plakina trilopha* | *Plakinidae* | Adult | Pltr_A_1 | ERX1233041 | 1257:10346.SS.8.1 |
| *Plakina trilopha* | *Plakinidae* | Adult | Pltr_A_2 | ERX1233042 | 1257:10346.SS.8.2 |
| *Plakina trilopha* | *Plakinidae* | Adult | Pltr_A_3 | ERX1233043 | 1257:10346.SS.8.3 |
| *Plakina trilopha* | *Plakinidae* | Adult | Pltr_A_4 | ERX1837188 | 2367:10793.10533.Taylor.WS.15.1 |
| *Plakina trilopha* | *Plakinidae* | Adult | Pltr_A_5 | ERX1837189 | 2367:10793.10533.Taylor.WS.15.11 |
| *Plakina trilopha* | *Plakinidae* | Adult | Pltr_A_6 | ERX1837190 | 2367:10793.10533.Taylor.WS.15.3 |
| *Plakina trilopha* | *Plakinidae* | Adult | Pltr_A_7 | ERX1837191 | 2367:10793.10533.Taylor.WS.15.7 |
| *Plakina trilopha* | *Plakinidae* | Not provided (Adult) | Pltr_A_8 | ERX1837192 | 2367:10793.10533.Taylor.WS.15.9 |
| *Plakina trilopha* | *Plakinidae* | Not provided (Adult) | Pltr_A_9 | ERX1839876 | 2370:10793.SS.8.1.1182306 |
| *Plakina trilopha* | *Plakinidae* | Not provided (Adult) | Pltr_A_10 | ERX1839877 | 2370:10793.SS.8.2.1181152 |
| *Plakina trilopha* | *Plakinidae* | Adult | Pltr_A_11 | ERX1839878 | 2370:10793.SS.8.3.1181204 |
| *Plakinastrella* sp. | *Plakinidae* | Adult | Plakinastrella_A | ERX1842799 | 2334:10793.10533.BDR049 |
| *Plakortis* sp. | *Plakinidae* | Not provided (Adult) | Pl_A_1 | ERX1839132 | 2370:10793.101.1181093 |
| *Plakortis* sp. | *Plakinidae* | Not provided (Adult) | Pl_A_2 | ERX1839134 | 2370:10793.103.1181271 |
| *Plakortis* sp. | *Plakinidae* | Not provided (Adult) | Pl_A_3 | ERX1839135 | 2370:10793.104.1182033 |
| *Plakortis simplex* | *Plakinidae* | Not provided (Adult) | Plsi_A_4 | ERX1836739 | 2368:10793.10533.PS1 |
| *Plakortis simplex* | *Plakinidae* | Not provided (Adult) | Plsi_A_5 | ERX1836740 | 2368:10793.10533.PS2 |
| *Plakortis simplex* | *Plakinidae* | Not provided (Adult) | Plsi_A_6 | ERX1836741 | 2368:10793.10533.PS3 |
| *Plakortis simplex* | *Plakinidae* | Not provided (Adult) | Plsi_A_7 | ERX1836742 | 2368:10793.10533.PS4 |
| *Plakortis halichondrioides* | *Plakinidae* | Not provided (Adult) | Plha_A_8 | ERX1836898 | 2367:10793.10533.PTL09.P15.XT |
| *Plakortis angulospiculatus* | *Plakinidae* | Not provided (Adult) | Plan_A_9 | ERX1837084 | 2367:10793.10533.SI06.157.XT |
| *Plakortis angulospiculatus* | *Plakinidae* | Not provided (Adult) | Plan_A_10 | ERX1837093 | 2367:10793.10533.SI06.197.XT |
| *Plakortis angulospiculatus* | *Plakinidae* | Not provided (Adult) | Plan_A_11 | ERX1837097 | 2367:10793.10533.SI06.211.XT |
| *Plakortis halichondrioides* | *Plakinidae* | Not provided (Adult) | Plha_A_12 | ERX1837139 | 2367:10793.10533.SI07.109.XT |
| *Plakortis sp.* | *Plakinidae* | Not provided (Adult) | Pl_A_13 | ERX1843064 | 2334:10793.10533.Saba..34 |
| *Plakortis sp.* | *Plakinidae* | Not provided (Adult) | Pl_A_14 | ERX1843123 | 2334:10793.10533.UH35 |
| *Plakortis sp.* | *Plakinidae* | Not provided (Adult) | Pl_A_15 | ERX1843124 | 2334:10793.10533.UH36 |
| *Plakortis sp.* | *Plakinidae* | Not provided (Adult) | Pl_A_16 | ERX1843125 | 2334:10793.10533.UH37 |
| *Plakortis sp.* | *Plakinidae* | Not provided (Adult) | Pl_A_17 | ERX1843127 | 2334:10793.10533.UH39 |
| *Plakortis halichondrioides* | *Plakinidae* | Not provided (Adult) | Plha_A_18 | ERX1843129 | 2334:10793.10533.UH40 |
| *Plakortis sp.* | *Plakinidae* | Not provided (Adult) | Pl_A_19 | ERX1839138 | 2370:10793.107.1182338 |
| *Plakortis sp.* | *Plakinidae* | Adult | Pl_A_20 | ERX1839146 | 2370:10793.111.1181115 |
| *Plakortis sp.* | *Plakinidae* | Adult | Pl_A_21 | ERX1839147 | 2370:10793.112.1181089 |
| *Plakortis sp.* | *Plakinidae* | Adult | Pl_A_22 | ERX1839149 | 2370:10793.114.1181513 |
| *Plakortis sp.* | *Plakinidae* | Adult | Pl_A_23 | ERX1839150 | 2370:10793.115.1181557 |
| *Plakortis sp.* | *Plakinidae* | Adult | Pl_A_24 | ERX1839153 | 2370:10793.118.1181649 |
| *Plakortis sp.* | *Plakinidae* | Adult | Pl_A_25 | ERX1839158 | 2370:10793.121.1181562 |
| *Plakortis sp.* | *Plakinidae* | Adult | Pl_A_26 | ERX1839159 | 2370:10793.122.1181248 |
| *Plakortis sp.* | *Plakinidae* | Adult | Pl_A_27 | ERX1839160 | 2370:10793.123.1181969 |
| *Plakortis sp.* | *Plakinidae* | Adult | Pl_A_28 | ERX1839161 | 2370:10793.124.1181117 |
| *Plakortis sp.* | *Plakinidae* | Not provided (Adult) | Pl_A_29 | ERX1839291 | 2370:10793.65.1182218 |
| *Plakortis sp.* | *Plakinidae* | Not provided (Adult) | Pl_A_30 | ERX1839292 | 2370:10793.66.1181812 |
| *Plakortis sp.* | *Plakinidae* | Not provided (Adult) | Pl_A_31 | ERX1839315 | 2370:10793.75.1181580 |
| *Plakortis sp.* | *Plakinidae* | Not provided (Adult) | Pl_A_32 | ERX1839316 | 2370:10793.76.1181800 |
| *Plakortis sp.* | *Plakinidae* | Not provided (Adult) | Pl_A_33 | ERX1839320 | 2370:10793.80.1182374 |
| *Plakortis sp.* | *Plakinidae* | Not provided (Adult) | Pl_A_34 | ERX1839323 | 2370:10793.83.1181372 |
| *Plakortis halichondrioides* | *Plakinidae* | Not provided (Adult) | Plha_A_35 | ERX1839325 | 2370:10793.85.1182278 |
| *Plakortis halichondrioides* | *Plakinidae* | Not provided (Adult) | Plha_A_36 | ERX1839326 | 2370:10793.86.1181915 |
| *Plakortis halichondrioides* | *Plakinidae* | Not provided (Adult) | Plha_A_37 | ERX1839327 | 2370:10793.87.1181122 |
| *Plakortis halichondrioides* | *Plakinidae* | Not provided (Adult) | Plha_A_38 | ERX1839328 | 2370:10793.88.1181685 |
| *Plakortis halichondrioides* | *Plakinidae* | Not provided (Adult) | Plha_A_39 | ERX1839329 | 2370:10793.89.1181419 |
| *Plakortis halichondrioides* | *Plakinidae* | Not provided (Adult) | Plha_A_40 | ERX1839330 | 2370:10793.90.1181233 |
| *Plakortis halichondrioides* | *Plakinidae* | Not provided (Adult) | Plha_A_41 | ERX1839331 | 2370:10793.91.1181725 |
| *Plakortis halichondrioides* | *Plakinidae* | Not provided (Adult) | Plha_A_42 | ERX1839332 | 2370:10793.92.1181744 |
| *Plakortis sp.* | *Plakinidae* | Not provided (Adult) | Pl_A_43 | ERX1839335 | 2370:10793.95.1181660 |
| *Plakortis sp.* | *Plakinidae* | Not provided (Adult) | Pl_A_44 | ERX1839339 | 2370:10793.99.1181308 |

SRA: Sequence Read Archive. ID: Identification.

**Table S2.** Sequence statistics and alpha diversity metrics of sponge-associated prokaryotic communities found in different life stages of *P. cyanorosea*, ambient seawater and sediment.

| **Sample** | **Read count** | **OTU number**^1^ | **Unique OTUs** | **Chao1** | **Shannon** |
| --- | --- | --- | --- | --- | --- |
| A1 | 319,054 | 4,109 | 651 | 5,499.563 | 3.051043 |
| A2 | 321,246 | 3,427 | 410 | 5,274.284 | 2.794318 |
| A3 | 322,045 | 3,196 | 210 | 5,883.159 | 2.640728 |
| L1 | 321,967 | 3,774 | 583 | 5,896.599 | 3.683668 |
| L2 | 321,807 | 3,678 | 544 | 5,575.336 | 3.275295 |
| L3 | 323,477 | 2,916 | 202 | 5,308.545 | 2.517719 |
| W1 | 320,078 | 3,794 | 665 | 4,787.231 | 1.858617 |
| W2 | 320,713 | 3,885 | 597 | 5,330.809 | 2.132883 |
| W3 | 320,772 | 2,304 | 1,174 | 2,533.738 | 1.765604 |
| S1 | 319,966 | 9,787 | 3,056 | 10,680.7 | 6.932373 |
| S2 | 316,827 | 8,951 | 2,034 | 10,512.12 | 6.311365 |
| S3 | 317,511 | 6,867 | 1,186 | 8,477.251 | 5.027134 |

^1^ For taxonomic classification, the read count was subsampled to the sample containing the fewest reads (n = 316,827).

**Table S3.** Statistical assessment of the alpha diversity metrics for the pairwise comparisons between two groups (sponge vs. environment; adults vs. larvae) by the *t*-tests and all groups by One-Way ANOVA.

| **Sample groups** | **OTU number** | **Chao1** | **Shannon** |
| --- | --- | --- | --- |
| Sponge vs. environment (*t*-test) | t = 6.7226  ***p* = 3.2751e-05** | t = 9.1661  ***p* = 1.7522e-06** | t = 7.0986  ***p* = 1.9968 e-05** |
| Adult vs. larvae  (*t*-test) | t = 20.139 | t = 50.491 | t = 16.828 |
|  | ***p* = 5.5812e-06** | ***p* = 5.7601e-08** | ***p* = 1.3548E-05** |
| All groups  (One-way ANOVA) | F = 22.19  ***p* = 0.000311** | F = 18.82  ***p* = 0.0005536** | F = 28.47  ***p* = 0.0001278** |

**Table S4.** Taxonomic classification of unique and uniquely shared OTUs found in each sample group or between combination of sample groups.

| Top OTU | Domain | Phylum | Class | Order | Family | Genus | Group |
| --- | --- | --- | --- | --- | --- | --- | --- |
| 205 | Bacteria | Proteobacteria | Gammaproteobacteria | Gammaproteobacteria_unclassified | Gammaproteobacteria_unclassified | Gammaproteobacteria_unclassified | A, L |
| 575 | Bacteria | Proteobacteria | Gammaproteobacteria | Alteromonodales | Ferrimonadaceae | *Ferrimonas* | A, L |
| 960 | Bacteria | Proteobacteria | Alphaproteobacteria | Alphaproteobacteria_unclassified | Alphaproteobacteria_unclassified | Alphaproteobacteria_unclassified | A, L |
| 1664 | Bacteria | Proteobacteria | Alphaproteobacteria | Alphaproteobacteria_unclassified | Alphaproteobacteria_unclassified | Alphaproteobacteria_unclassified | A, L |
| 3156 | Bacteria | Proteobacteria | Gammaproteobacteria | Vibrionales | Vibrionaceae | Vibrionaceae_unclassified | A, L |
| 167 | Bacteria | Proteobacteria | Alphaproteobacteria | Rhizobiales | Phyllobacteriaceae | *Lentilitoribacter* | W, S |
| 775 | Bacteria | Proteobacteria | Alphaproteobacteria | Rhodobacterales | Rhodobacteraceae | Rhodobacteraceae_unclassified | W, S |
| 104 | Bacteria | Proteobacteria | Gammaproteobacteria | Alteromonodales | Pseudoalteromonodaceae | *Psychrosphaera* | A, L, W |
| 309 | Bacteria | Proteobacteria | Gammaproteobacteria | Enterobacteriales | Enterobacteriaceae | Enterobacteriaceae_unclassified | A, L, S |
| 1045 | Archaea | Thaumarchaeota | - | Nitrosopumilales | Nitrosopumilaceae | Nitrosopumilus_unclassified | A, L, S |
| 2206 | Bacteria | Proteobacteria | Gammaproteobacteria | Oceanospirillales | Halomonadaceae | *Halomonas* | A, L, S |
| 52 | Bacteria | Bacteroidetes | Bacteroidiia | Bacteroidales | Bacteroidaceae | *Bacteroides* | L, W, S |
| 67 | Bacteria | Bacteroidetes | Bacteroidiia | Bacteroidales | Bacteroidaceae | *Bacteroides* | L, W, S |

A: adults, L: larvae; W: water; S: sediment.

**Table S5.** Taxonomic classification and relative abundance of the top30 OTUs detected in the different life stages of *P. cyanorosea* and environmental samples.

| Top OTU | Domain | Phylum | Class | Order | Family | Genus | Relative abundance |
| --- | --- | --- | --- | --- | --- | --- | --- |
| 1 | Bacteria | Proteobacteria | Gammaproteobacteria | Enterobacterales | Enterobacteriaceae | Enterobacteriaceae_unclassified | 16.60% |
| 2 | Bacteria | Proteobacteria | Epsilonproteobacteria | Campylobacterales | Campylobacteraceae | *Arcobacter* | 9.95% |
| 3 | Bacteria | Proteobacteria | Gammaproteobacteria | Alteromonodales | Pseudoalteromonodaceae | *Pseudoalteromonas* | 9.39% |
| 4 | Bacteria | Proteobacteria | Gammaproteobacteria | Vibrionales | Vibrionaceae | *Vibrio* | 4.76% |
| 5 | Archaea | Thaumarchaeota | - | Nitrosopumilales | Nitrosopumilaceae | Nitrosopumilus_unclassified | 3.37% |
| 6 | Bacteria | Proteobacteria | Alphaproteobacteria | Alphaproteobacteria_unclassified | Alphaproteobacteria_unclassified | Alphaproteobacteria_unclassified | 3.29% |
| 7 | Bacteria | Proteobacteria | Gammaproteobacteria | Vibrionales | Vibrionaceae | *Vibrio* | 3.22% |
| 8 | Bacteria | Proteobacteria | Alphaproteobacteria | Alphaproteobacteria_unclassified | Alphaproteobacteria_unclassified | Alphaproteobacteria_unclassified | 3.16% |
| 9 | Bacteria | Proteobacteria | Gammaproteobacteria | Pseudomonodales | Pseudomonodaceae | *Pseudomonas* | 3.12% |
| 10 | Bacteria | Proteobacteria | Gammaproteobacteria | Gammaproteobacteria_unclassified | Gammaproteobacteria_unclassified | Gammaproteobacteria_unclassified | 2.07% |
| 11 | Bacteria | Proteobacteria | Proteobacteria_unclassified | Proteobacteria_unclassified | Proteobacteria_unclassified | Proteobacteria_unclassified | 1.53% |
| 12 | Bacteria | Proteobacteria | Gammaproteobacteria | Alteromonodales | Alteromonadaceae | *Alteromonas* | 1.06% |
| 13 | Bacteria | Proteobacteria | Epsilonproteobacteria | Campylobacterales | Campylobacteraceae | *Arcobacter* | 0.81% |
| 14 | Bacteria | Proteobacteria | Gammaproteobacteria | Oceanospirillales | Oceanospirillaceae | *Marinomonas* | 0.80% |
| 15 | Bacteria | Proteobacteria | Alphaproteobacteria | Alphaproteobacteria_unclassified | Alphaproteobacteria_unclassified | Alphaproteobacteria_unclassified | 0.68% |
| 16 | Bacteria | Proteobacteria | Proteobacteria_unclassified | Proteobacteria_unclassified | Proteobacteria_unclassified | Proteobacteria_unclassified | 0.65% |
| 17 | Bacteria | Proteobacteria | Alphaproteobacteria | Alphaproteobacteria_unclassified | Alphaproteobacteria_unclassified | Alphaproteobacteria_unclassified | 0.57% |
| 18 | Bacteria | Proteobacteria | Proteobacteria_unclassified | Proteobacteria_unclassified | Proteobacteria_unclassified | Proteobacteria_unclassified | 0.55% |
| 19 | Bacteria | Proteobacteria | Gammaproteobacteria | Alteromonodales | Pseudoalteromonodaceae | *Psychrosphaera* | 0.51% |
| 20 | Bacteria | Proteobacteria | Gammaproteobacteria | Chromatiales | Chromatiales_unclassified | Chromatiales_unclassified | 0.51% |
| 21 | Bacteria | Proteobacteria | Alphaproteobacteria | Rhodospirillales | Rhodospirillaceae | *Thalassospira* | 0.45% |
| 22 | Bacteria | Proteobacteria | Alphaproteobacteria | Rhodospirillales | Rhodospirillaceae_unclassified | Rhodospirillaceae_unclassified | 0.37% |
| 23 | Bacteria | Proteobacteria | Alphaproteobacteria | Alphaproteobacteria_unclassified | Alphaproteobacteria_unclassified | Alphaproteobacteria_unclassified | 0.36% |
| 24 | Bacteria | Proteobacteria | Gammaproteobacteria | Alteromonodales | Colwelliaceae | *Thalassotalea* | 0.36% |
| 25 | Bacteria | Proteobacteria | Proteobacteria_unclassified | Proteobacteria_unclassified | Proteobacteria_unclassified | Proteobacteria_unclassified | 0.36% |
| 26 | Bacteria | Proteobacteria | Gammaproteobacteria | Vibrionales | Vibrionaceae | *Vibrio* | 0.36% |
| 27 | Bacteria | Proteobacteria | Alphaproteobacteria | Alphaproteobacteria_unclassified | Alphaproteobacteria_unclassified | Alphaproteobacteria_unclassified | 0.34% |
| 28 | Bacteria | Proteobacteria | Gammaproteobacteria | Chromatiales | Chromatiales_unclassified | Chromatiales_unclassified | 0.33% |
| 29 | Bacteria | Proteobacteria | Gammaproteobacteria | Alteromonodales | Alteromonadaceae | Alteromonadaceae_unclassified | 0.32% |
| 30 | Bacteria | Proteobacteria | Gammaproteobacteria | Alteromonodales | Colwelliaceae | *Thalassotalea* | 0.32% |

**Table S6.** List of sponge hosts containing the significantly enriched closest subOTUs relatives of the top30 and 13 unique and uniquely shared OTUs found in *P. cyanorosea* and environmental samples.

| **Sponge host** | **total subOTUs / total of sequences in the host** | **binomial_p** | **ranksum_p** |
| --- | --- | --- | --- |
| *Niphates digitalis* | (34/175) | 0 | 0.018585 |
| *Haliclona maravillosa* | (12/70) | 0.000188 | 0.109629 |
| Porifera, branching, at taxonomist | (6/35) | 0.007587 | 0.090782 |
| *Haliclona walentinae* | (46/315) | 0 | 0.00003 |
| *Haliclona mediterranea* | (25/175) | 0.000003 | 0.030318 |
| *Stelletta maori* | (14/105) | 0.000835 | 0.003251 |
| *Siphonodictyon* (Aka) *coralliphagum* | (14/105) | 0.000835 | 0.003251 |
| *Haliclona indistincta* | (27/210) | 0.000009 | 0.031415 |
| Porifera, Luffariella like, at taxonomist | (9/70) | 0.008519 | 0.068375 |
| *Haliclona cinerea* | (9/70) | 0.008519 | 0.098875 |
| Dysidea etheria | (42/350) | 0 | 0.111135 |
| *Halichondria phakellioides* | (21/175) | 0.000223 | 0.293896 |
| Porifera | (1039/8750) | 0 | 0 |
| *Spheciospongia vagabunda* | (16/140) | 0.001971 | 0.262358 |
| *Callyspongia siphonella* | (4/35) | 0.098328 | 0.784547 |
| Porifera, light yellow, at taxonomist | (4/35) | 0.098328 | 0.474135 |
| *Cliona* sp. | (4/35) | 0.098328 | 0.130579 |
| *Iotrochota* sp. | (18/175) | 0.003455 | 0.62476 |
| *Scopalina ruetzleri* | (18/175) | 0.003455 | 0.416129 |
| *Myxilla* sp. | (21/210) | 0.002373 | 0.233438 |
| *Petrosia (Strongylophora) hartmani* | (7/70) | 0.062866 | 0.431859 |
| Porifera, off-white lump, at taxonomist | (7/70) | 0.062866 | 0.154567 |
| *Terpios hoshinota* | (41/420) | 0.000054 | 0.000141 |
| *Mycale laxissima* | (102/1155) | 0 | 0.002075 |
| *Cliona celata complex* | (21/245) | 0.013298 | 0.025889 |
| *Darwinella oxeata* | (9/105) | 0.084069 | 0.012285 |
| *Cliona celata* | (41/490) | 0.001265 | 0.001296 |
| *Mycale laevis* | (29/350) | 0.006714 | 0.473805 |
| *Dysidea fragilis* | (57/700) | 0.000339 | 0.027982 |
| *Clathrina coriacea* | (17/210) | 0.038419 | 0.000018 |
| *Haliclona fascigera* | (17/210) | 0.038419 | 0.408548 |
| *Raspaciona aculeata* | (16/210) | 0.067663 | 0.562554 |
| *Tetrapocillon minor* | (16/210) | 0.067663 | 0.103245 |
| *Phorbas fictitius* | (60/805) | 0.00204 | 0.715297 |
| *Axinella infundibuliformis* | (31/420) | 0.023751 | 0 |
| *Phakellia fusca* | (10/140) | 0.171129 | 0.011207 |
| *Chalinula* sp. | (10/140) | 0.171129 | 0.05965 |
| *Plakina trilopha* | (17/245) | 0.116704 | 0.008408 |
| *Placospongia intermedia* | (17/245) | 0.116704 | 0.030559 |
| *Niphates erecta* | (24/350) | 0.081755 | 1 |
| *Suberites carnosus* | (7/105) | 0.280052 | 0.007601 |
| *Xestospongia* sp. | (88/1330) | 0.006954 | 0.117877 |
| *Dysidea* sp. | (37/560) | 0.060421 | 0.002989 |
| *Dysidea avara* | (131/2030) | 0.00302 | 0.000281 |
| *Cliona delitrix* | (109/1715) | 0.009465 | 0.242899 |
| *Axinella rugosa* | (11/175) | 0.270917 | 0.009585 |
| *Crella incrustans* | (11/175) | 0.270917 | 0.009756 |
| *Amphimedon compressa* | (59/945) | 0.057817 | 0.621331 |
| *Crambe crambe* | (113/1820) | 0.015682 | 0.010719 |
| *Leucetta* sp. | (21/350) | 0.238575 | 0.000019 |
| *Plakortis* sp. | (62/1085) | 0.174523 | 0 |
| *Latrunculia* sp*.* | (20/350) | 0.316863 | 0.000373 |
| *Phakellia ventilabrum* | (6/105) | 0.43791 | 0.001779 |
| *Stelletta aremaria* | (6/105) | 0.43791 | 0.005841 |
| *Ptilocaulis walpersi* | (6/105) | 0.43791 | 0.001801 |
| *Haliclona oculata* | (48/875) | 0.297607 | 0.00022 |
| *Clathrina sp.* | (17/315) | 0.423922 | 0.001636 |
| *Geodia barretti* | (178/4095) | 0.983146 | 0 |
| *Oscarella lobularis* | (19/455) | 0.83027 | 0.048711 |
| *Ircinia strobilina* | (43/1085) | 0.959619 | 0.000011 |

binomial_p: *p* values of presence/abnormal binomial test; ranksum_p: *p* values of relative frequency-based ranksum test

**Table S7.** Alpha diversity metrics obtained for the Homoscleromorph sponges and environmental samples in the global analyses.

| **Sample (ID)** | **OTU number** | **Chao1** | **Shannon** |
| --- | --- | --- | --- |
| *Oscarella lobularis* adult 1 (Oslo_A_1) | 1,431 | 4,009.8426 | 3.1210057 |
| *Oscarella lobularis* adult 2 (Oslo_A_2) | 2,249 | 5,319.2229 | 3.2663360 |
| *Oscarella lobularis* adult 3 (Oslo_A_3) | 2,466 | 5,388.7494 | 3.3656968 |
| *Oscarella lobularis* adult 4 (Oslo_A_4) | 3,110 | 5,969.2373 | 3.1584959 |
| *Oscarella lobularis* adult 5 (Oslo_A_5) | 4,165 | 7,963.4488 | 3.6382402 |
| *Oscarella lobularis* adult 6 (Oslo_A_6) | 5,202 | 8,451.6098 | 3.5026290 |
| *Oscarella lobularis* adult 7 (Oslo_A_7) | 1,026 | 1,650.5939 | 1.1514072 |
| *Oscarella lobularis* adult 8 (Oslo_A_8) | 1,217 | 1,898.1805 | 0.7683934 |
| *Oscarella lobularis*  larvae 1 (Oslo_L_1) | 62 | 603.5000 | 0.5710165 |
| *Oscarella lobularis* larvae 2 (Oslo_L_2) | 70 | 670.0000 | 1.7001368 |
| *Oscarella lobularis* larvae 3 (Oslo_L_3) | 70 | 350.9000 | 1.9694119 |
| *Oscarella lobularis* larvae 4 (Oslo_L_4) | 770 | 1,561.2266 | 0.4531655 |
| *Oscarella lobularis* larvae 5 (Oslo_L_5) | 5,084 | 8,766.5467 | 3.7988279 |
| *Oscarella lobularis* larvae 6 (Oslo_L_6) | 48 | 288.6667 | 1.5327313 |
| *Oscarella lobularis* larvae 7 (Oslo_L_7) | 62 | 206.0000 | 0.8523686 |
| *Oscarella lobularis*  larvae 8 (Oslo_L_8) | 151 | 838.6818 | 1.9992836 |
| *Oscarella lobularis* larvae 9 (Oslo_L_9) | 67 | 667.0000 | 4.0387779 |
| *Pseudocorticium jarrei* adult 1 (Psja_A_1) | 2,275 | 11,930.3820 | 4.1542351 |
| *Pseudocorticium jarrei* adult 2 (Psja_A_2) | 2,406 | 11,077.5372 | 4.1156024 |
| *Pseudocorticium jarrei* adult 3 (Psja_A_3) | 2,645 | 13,172.5766 | 4.1537396 |
| *Pseudocorticium jarrei* adult 4 (Psja_A_4) | 2,275 | 11,930.3820 | 4.1542351 |
| *Pseudocorticium jarrei* adult 5 (Psja_A_5) | 2,406 | 11,077.5372 | 4.1156024 |
| *Pseudocorticium jarrei* adult 6 (Psja_A_6) | 2,645 | 13,172.5766 | 4.1537396 |
| *Corticium candelabrum* adult 1 (Coca_A_1) | 1,985 | 5,292.5674 | 3.1904391 |
| *Corticium candelabrum* adult 2 (Coca_A_2) | 2,581 | 6,048.9402 | 3.3460624 |
| *Corticium candelabrum* adult 3 (Coca_A_3) | 1,116 | 3,102.4862 | 3.3571012 |
| *Corticium candelabrum* larvae 1 (Coca_L_1) | 7,806 | 16,290.0930 | 3.5286213 |
| *Corticium candelabrum* larvae 2 (Coca_L_2) | 5,958 | 13,434.1194 | 3.5245868 |
| *Corticium candelabrum* larvae 3 (Coca_L_3) | 11,240 | 17,268.7755 | 3.5328529 |
| *Corticium candelabrum* larvae 4 (Coca_L_4) | 48 | 150.4000 | 2.5299276 |
| *Corticium* sp. larvae 5 (Coca_L_5) | 1,625 | 4,709.1604 | 3.5440687 |
| *Corticium* sp. larvae 6 (Coca_L_6) | 2,551 | 5,888.9142 | 3.5707275 |
| *Plakina trilopha* adult 1 *(*Pltr_A_1) | 2,103 | 9,198.2968 | 3.7196725 |
| *Plakina trilopha* adult 2 *(*Pltr_A_2) | 4,579 | 18,187.0890 | 3.8565929 |
| *Plakina trilopha* adult 3 *(*Pltr_A_3) | 2 | 3.0000 | 0.6931472 |
| *Plakina trilopha* adult 4 *(*Pltr_A_4) | 321 | 624.3426 | 2.4310157 |
| *Plakina trilopha* adult 5 *(*Pltr_A_5) | 266 | 642.4706 | 2.1454938 |
| *Plakina trilopha* adult 6 *(*Pltr_A_6) | 206 | 442.7427 | 1.8650361 |
| *Plakina trilopha* adult 7 *(*Pltr_A_7) | 241 | 645.4459 | 3.5048636 |
| *Plakina trilopha* adult 8 *(*Pltr_A_8) | 265 | 688.2903 | 2.8789016 |
| *Plakina trilopha* adult 9 *(*Pltr_A_9) | 2,103 | 9,198.2968 | 3.7196725 |
| *Plakina trilopha* adult 10 *(*Pltr_A_10) | 4,579 | 18,187.0890 | 3.8565929 |
| *Plakina trilopha* adult 11 *(*Pltr_A_11) | 2 | 3.0000 | 0.6931472 |
| *Plakinastrella* sp. adult 1 (Plakinastrella_A) | 877 | 1,680.3878 | 3.3643673 |
| *Plakortis* sp. adult 1 (Pl_A_1) | 1,462 | 4,141.5052 | 3.5231247 |
| *Plakortis* sp. adult 2 (Pl_A_2) | 1,352 | 4,429.3535 | 3.3937394 |
| *Plakortis* sp. adult 3 (Pl_A_3) | 1,570 | 3,898.6761 | 3.7722862 |
| *Plakortis simplex*  adult 4 (Plsi_A_4) | 9,587 | 16,896.9154 | 4.5649517 |
| *Plakortis simplex*  adult 5 (Plsi_A_5) | 7,467 | 16,083.3166 | 4.5067665 |
| *Plakortis simplex*  adult 6 (Plsi_A_6) | 7,729 | 15,663.8520 | 4.4318159 |
| *Plakortis simplex*  adult 7 (Plsi_A_7) | 5,131 | 12,874.2099 | 4.4368834 |
| *Plakortis halichondrioides* adult 8 *(*Plha_A_8) | 2,842 | 7,524.8360 | 4.5902972 |
| *Plakortis angulospiculatus* adult 9 *(*Plan_A_9) | 3,218 | 9,108.5732 | 4.7525796 |
| *Plakortis angulospiculatus* adult 10 *(*Plan_A_10) | 3,502 | 7,853.0375 | 4.8317922 |
| *Plakortis angulospiculatus* adult 11 *(*Plan_A_11) | 3,591 | 8,143.0558 | 4.7681627 |
| *Plakortis halichondrioides* adult 12 *(*Plha_A_12) | 5,032 | 10,047.7462 | 4.6516000 |
| *Plakortis* sp. adult 13 (Pl_A_13) | 6,145 | 11,255.5856 | 4.6635390 |
| *Plakortis* sp. adult 14 (Pl_A_14) | 3,767 | 7,917.1911 | 4.0579256 |
| *Plakortis* sp. adult 15 (Pl_A_15) | 2,892 | 7,128.8258 | 4.1220848 |
| *Plakortis* sp. adult 16 (Pl_A_16) | 2,909 | 6,817.6969 | 4.4823797 |
| *Plakortis* sp. adult 17 (Pl_A_17) | 1,833 | 5,015.6614 | 3.9247173 |
| *Plakortis halichondrioides* adult 18 *(*Plha_A_18) | 3,976 | 8,933.8401 | 4.6044528 |
| *Plakortis* sp. adult 19 (Pl_A_19) | 1,528 | 3,913.6338 | 3.5045195 |
| *Plakortis* sp. adult 20 (Pl_A_20) | 1,483 | 4,010.5099 | 3.8170670 |
| *Plakortis* sp. adult 21 (Pl_A_21) | 1,646 | 4,425.3578 | 4.0715489 |
| *Plakortis* sp. adult 22 (Pl_A_22) | 1,336 | 3,846.4740 | 3.4613604 |
| *Plakortis* sp. adult 23 (Pl_A_23) | 1,617 | 4,056.3091 | 4.0771184 |
| *Plakortis* sp. adult 24 (Pl_A_24) | 1,363 | 4,072.1801 | 3.7933272 |
| *Plakortis* sp. adult 25 (Pl_A_25) | 2,176 | 4,137.1541 | 4.3687383 |
| *Plakortis* sp. adult 25 (Pl_A_26) | 1,927 | 3,740.1201 | 4.3641886 |
| *Plakortis* sp. adult 27 (Pl_A_27) | 1,430 | 3,533.5804 | 4.4403117 |
| *Plakortis* sp. adult 28 (Pl_A_28) | 1,238 | 3,163.7031 | 4.3064894 |
| *Plakortis* sp. adult 29 (Pl_A_29) | 1,239 | 3,396.5468 | 3.6917325 |
| *Plakortis* sp. adult 30 (Pl_A_30) | 1,044 | 3,013.0947 | 3.7864791 |
| *Plakortis* sp. adult 31 (Pl_A_31) | 1,590 | 4,100.6688 | 3.9378889 |
| *Plakortis* sp. adult 32 (Pl_A_32) | 1,273 | 3,939.6223 | 4.0766805 |
| *Plakortis* sp. adult 33 (Pl_A_33) | 1,710 | 4,212.1004 | 3.7667245 |
| *Plakortis* sp. adult 34 (Pl_A_34) | 1,360 | 4,754.0802 | 4.1356698 |
| *Plakortis halichondrioides* adult 35 *(*Plha_A_35) | 1,738 | 5,638.3218 | 4.5733863 |
| *Plakortis halichondrioides* adult 36 *(*Plha_A_36) | 1,237 | 4,071.2480 | 4.6728318 |
| *Plakortis halichondrioides* adult 37 *(*Plha_A_37) | 1,773 | 4,983.7456 | 4.2423298 |
| *Plakortis halichondrioides* adult 38 *(*Plha_A_38) | 2,000 | 5,686.7972 | 4.6198143 |
| *Plakortis halichondrioides* adult 39 *(*Plha_A_39) | 1,917 | 5,326.1654 | 4.4715148 |
| *Plakortis halichondrioides* adult 40 *(*Plha_A_40) | 1,306 | 4,015.7782 | 4.1505322 |
| *Plakortis halichondrioides* adult 41 *(*Plha_A_41) | 1,942 | 5,428.1250 | 4.4457059 |
| *Plakortis halichondrioides* adult 42 *(*Plha_A_42) | 1,977 | 5,652.9217 | 4.7722561 |
| *Plakortis* sp. adult 43 (Pl_A_43) | 1,602 | 3,897.3407 | 3.7696285 |
| *Plakortis* sp. adult 44 (Pl_A_44) | 1,436 | 4,465.7273 | 3.8258726 |
| A1 | 1,353 | 2,261.7189 | 2.5959478 |
| A2 | 1,314 | 2,124.0205 | 2.6134654 |
| A3 | 1,643 | 3,387.7120 | 2.4611674 |
| L1 | 3,545 | 5,078.0972 | 3.5919312 |
| L2 | 2,806 | 4,300.4606 | 3.0307319 |
| L3 | 2,430 | 4,278.5790 | 2.3125226 |
| W1 | 2,492 | 3,361.7172 | 1.7510816 |
| W2 | 2,651 | 3,665.7172 | 2.0097367 |
| W3 | 1,388 | 2,092.1799 | 1.6407726 |
| S1 | 9,633 | 10,415.7769 | 6.7074965 |
| S2 | 7,525 | 8,522.1285 | 6.0107880 |
| S3 | 5,303 | 6,469.2867 | 4.7538053 |

ID: Identification in accordance with the Table S1.

| 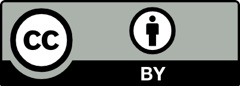 | © 2020 by the authors. Submitted for possible open access publication under the terms and conditions of the Creative Commons Attribution (CC BY) license (http://creativecommons.org/licenses/by/4.0/). |
| --- | --- |
