# Supplementary figures and images for: Not That Close to Mommy: Horizontal Transmission Seeds the Microbiome Associated with the Marine Sponge Plakina cyanorosea
Source: Microorganisms. 2020 Dec 12;8(12):1978. doi: 10.3390/microorganisms8121978 (PMC7764410; doi:10.3390/microorganisms8121978)

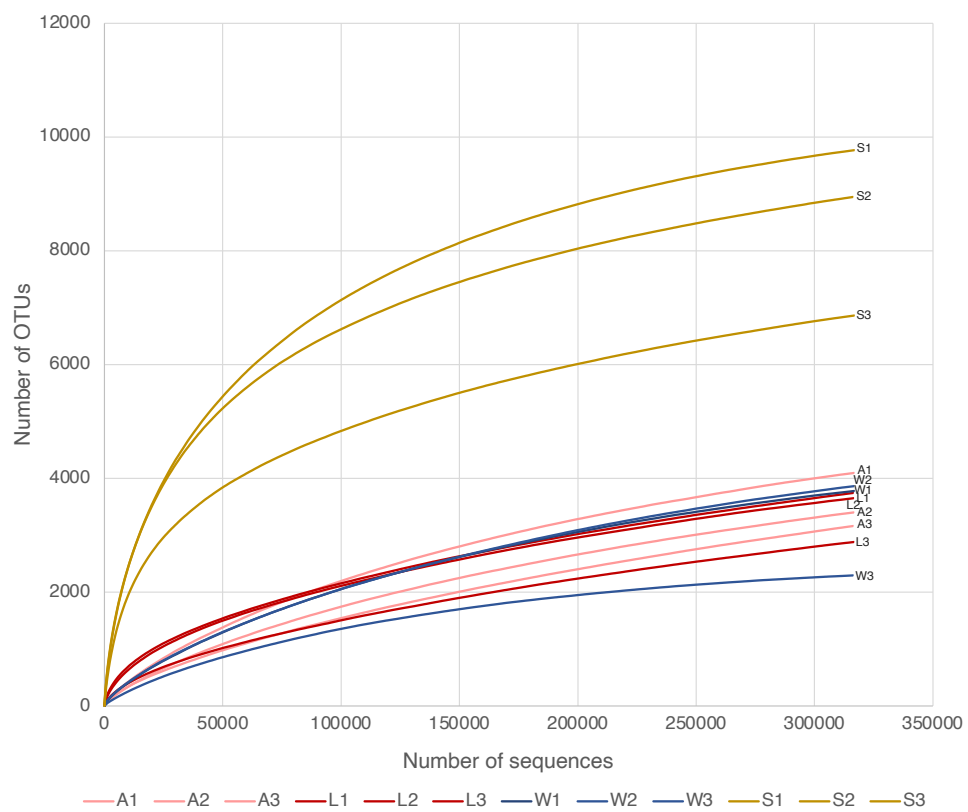

Supplement: Supplementary file 1 [file microorganisms-08-01978-s001.zip › Fig_S1.pdf]

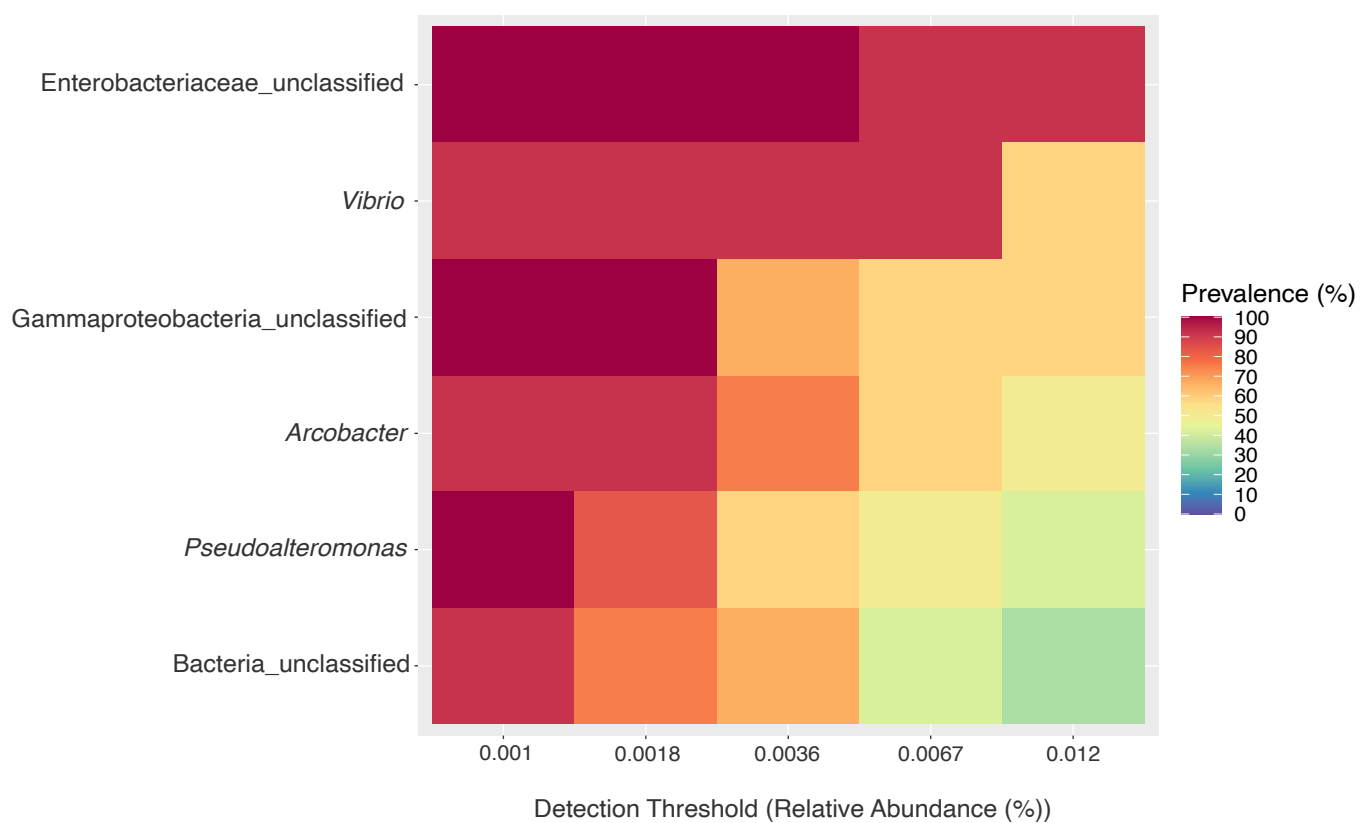

Supplement: Supplementary file 1 [file microorganisms-08-01978-s001.zip › Fig_S2.pdf]

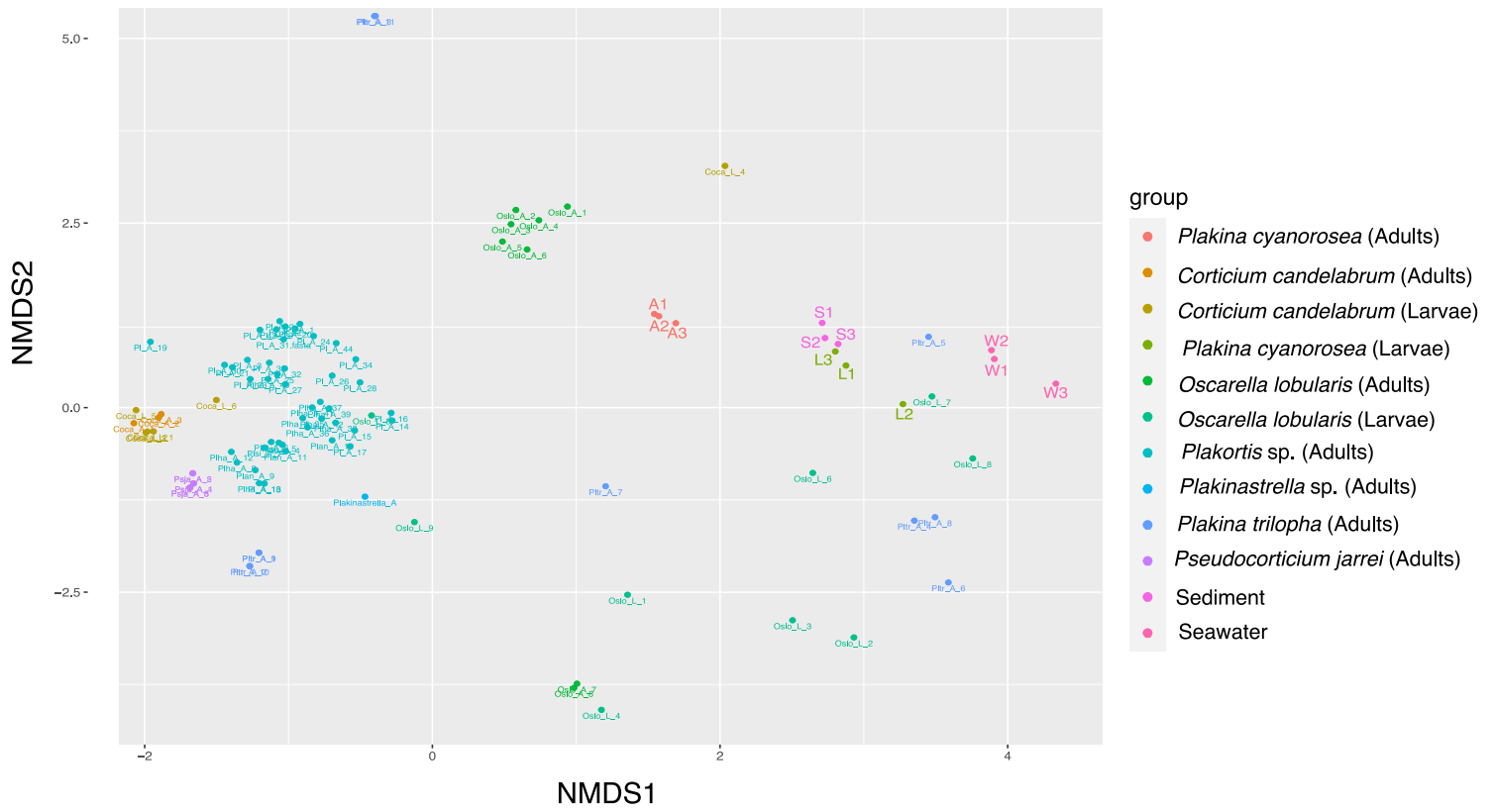

Supplement: Supplementary file 1 [file microorganisms-08-01978-s001.zip › Fig_S3.pdf]

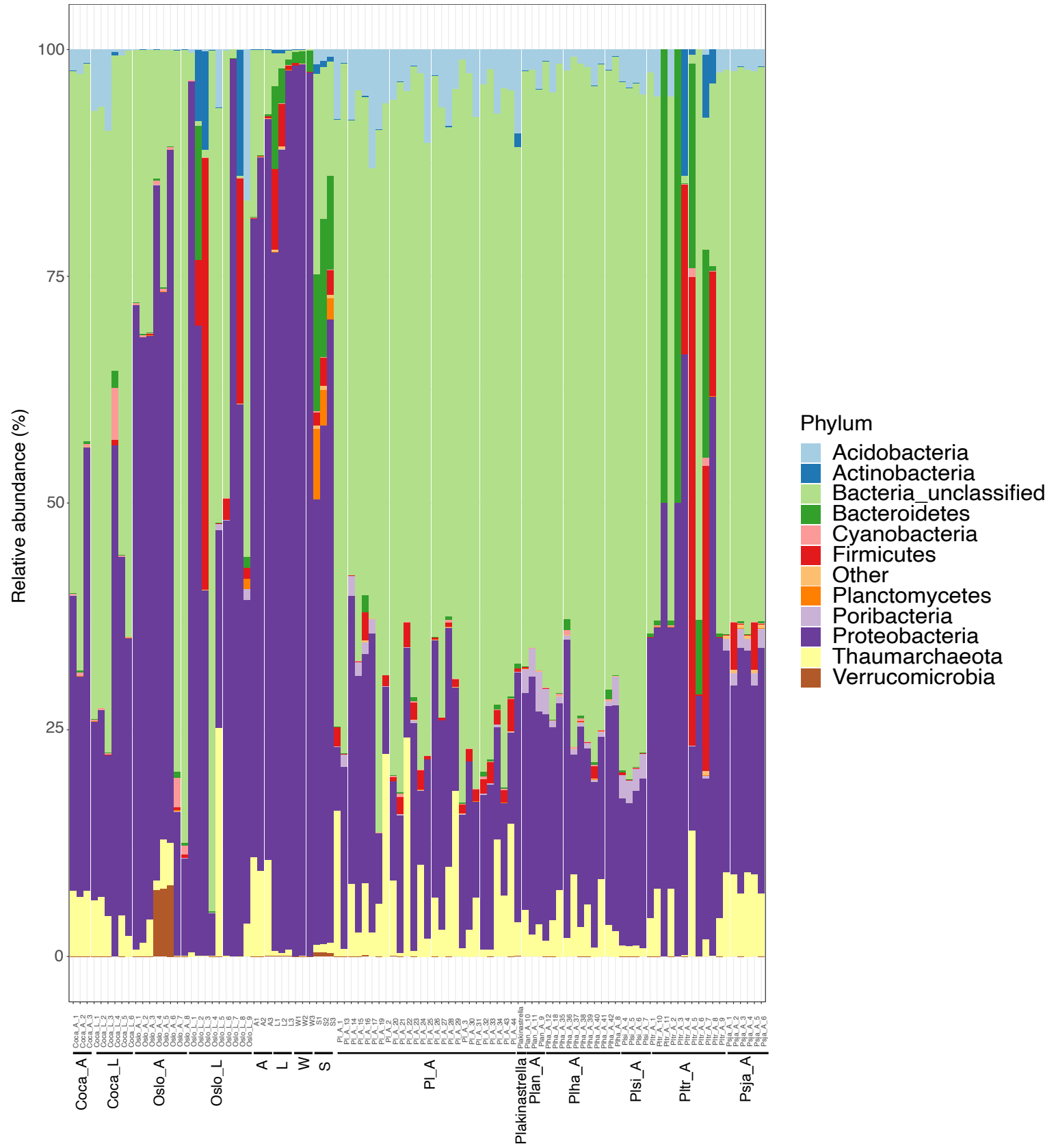

Supplement: Supplementary file 1 [file microorganisms-08-01978-s001.zip › Fig_S4.pdf]

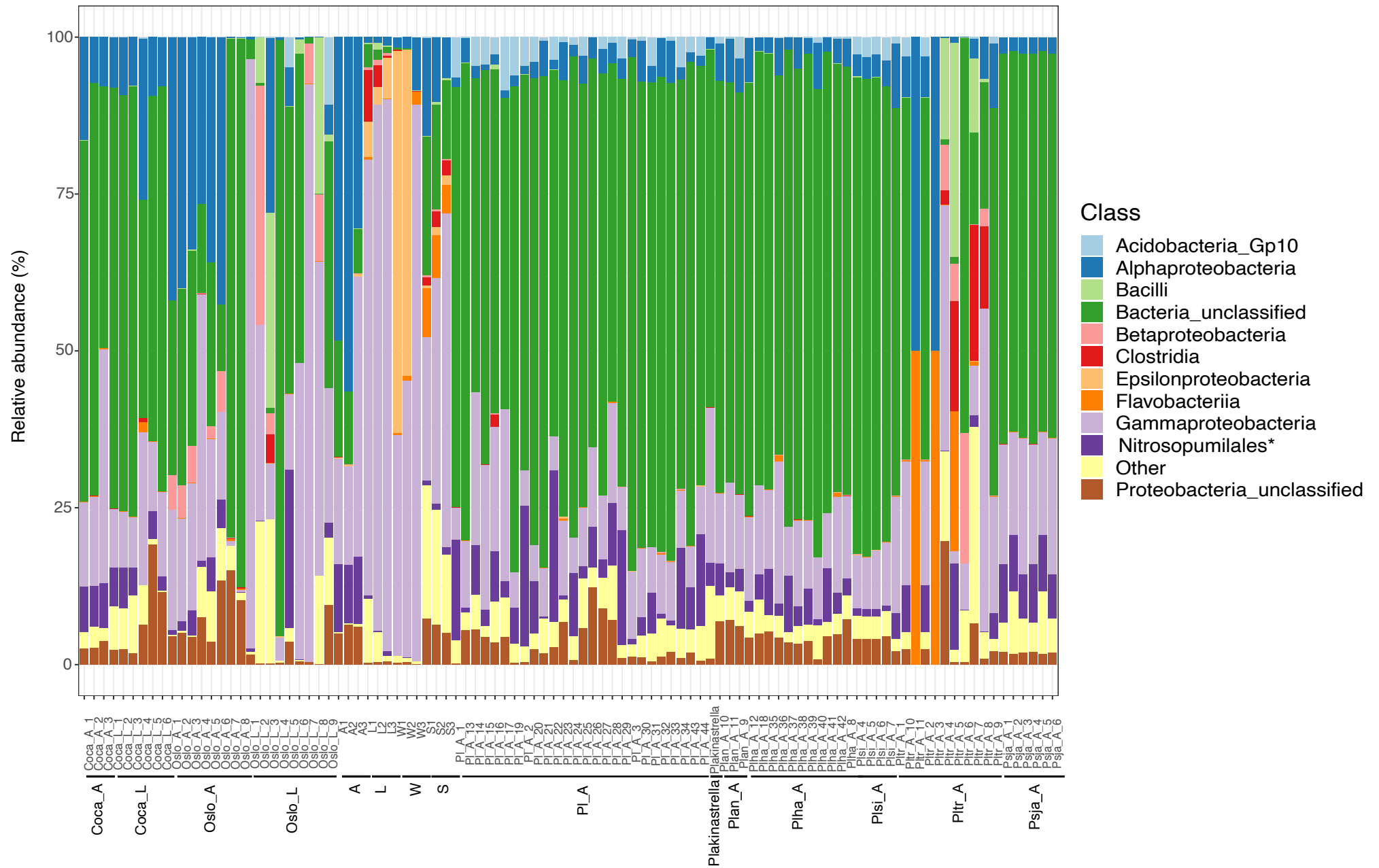

Supplement: Supplementary file 1 [file microorganisms-08-01978-s001.zip › Fig_S5.pdf]

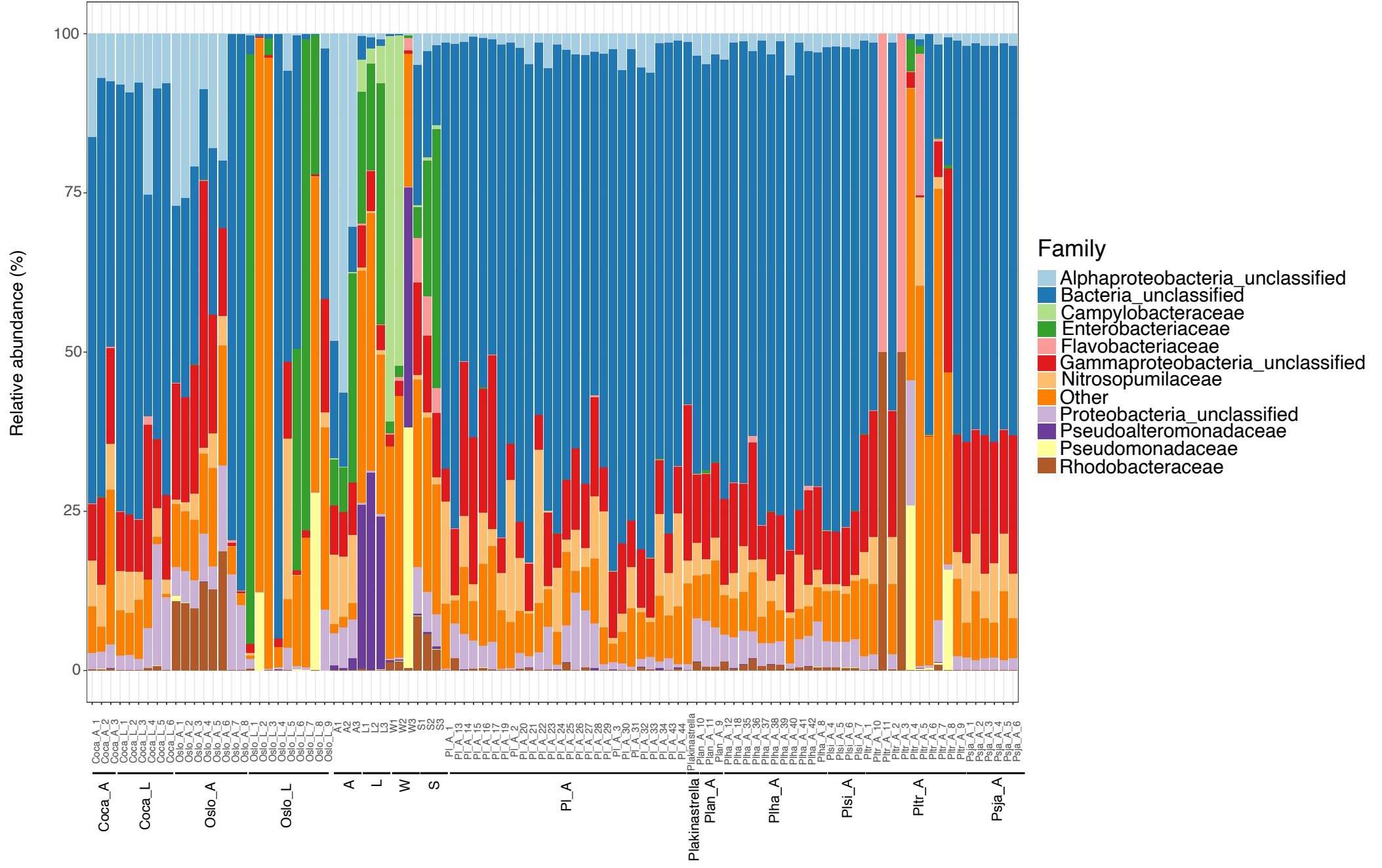

Supplement: Supplementary file 1 [file microorganisms-08-01978-s001.zip › Fig_S6.pdf]
